# Downregulation of *MTSS1* in acute myeloid leukemia is associated with a poor prognosis, chemotherapy resistance, and disease aggressiveness

**DOI:** 10.1038/s41375-021-01224-2

**Published:** 2021-03-29

**Authors:** Alexander Michael Grandits, Chi Huu Nguyen, Angela Schlerka, Hubert Hackl, Heinz Sill, Julia Etzler, Elizabeth Heyes, Dagmar Stoiber, Florian Grebien, Gerwin Heller, Rotraud Wieser

**Affiliations:** 1 Division of Oncology, Department of Medicine I, Medical University of Vienna, Vienna, Austria; 2 Comprehensive Cancer Center, Vienna, Austria; 3 Institute of Bioinformatics, Biocenter, Medical University of Innsbruck, Innsbruck, Austria; 4 Division of Hematology, Medical University of Graz, Graz, Austria; 5 Institute for Medical Biochemistry, University of Veterinary Medicine, Vienna, Austria; 6 Division of Pharmacology, Department of Pharmacology, Physiology and Microbiology, Karl Landsteiner University of Health Sciences, Krems, Austria

**Keywords:** Acute myeloid leukaemia, Translational research

## Abstract

Despite recent approval of targeted drugs for acute myeloid leukemia (AML) therapy, chemotherapy with cytosine arabinoside and anthracyclines remains an important pillar of treatment. Both primary and secondary resistance are frequent and associated with poor survival, yet the underlying molecular mechanisms are incompletely understood. In previous work, we identified genes deregulated between diagnosis and relapse of AML, corresponding to therapy naïve and resistant states, respectively. Among them was *MTSS1*, whose downregulation is known to enhance aggressiveness of solid tumors. Here we show that low *MTSS1* expression at diagnosis was associated with a poor prognosis in AML. *MTSS1* expression was regulated by promoter methylation, and reduced by cytosine arabinoside and the anthracycline daunorubicin. Experimental downregulation of *MTSS1* affected the expression of numerous genes. It induced the DNA damage response kinase WEE1, and rendered human AML cell lines more resistant to cytosine arabinoside, daunorubicin, and other anti-cancer drugs. *Mtss1* knockdown in murine *MLL-AF9*-driven AML substantially decreased disease latency, and increased leukemic burden and ex vivo chemotherapy resistance. In summary, low *MTSS1* expression represents a novel factor contributing to disease aggressiveness, therapy resistance, and poor outcome in AML.

## Background

Acute myeloid leukemia (AML) is caused by mutations and aberrant gene expression patterns emerging in hematopoietic stem and progenitor cells [1, 2]. Until recently, systemic chemotherapy consisting of cytosine arabinoside (araC) and an anthracycline (e.g., daunorubicin, DNR), followed by hematopoietic stem cell transplantation in a substantial number of patients, represented the only potentially curative therapeutic approach for patients with AML [3]. Novel targeted therapies were recently approved, but are available only for defined subsets of patients [3, 4]. Furthermore, they are increasingly used in combination with conventional cytotoxic drugs [4], so that these remain important pillars of AML treatment. With standard chemotherapy, complete remissions are achieved in more than half of all cases, but relapse occurs at a high rate [5, 6]. Even though relapse, which is caused by and associated with secondary resistance, and primary resistance are the main causes of death in AML, the molecular and genetic basis for therapy refractoriness in this disease remains incompletely understood. Many different genetic alterations were found to be newly acquired at relapse, yet very few of these were gained at this stage in a recurrent manner [7]. In contrast, a large number of genes were consistently and significantly deregulated between diagnosis and relapse of AML [8]. The impact of *SOCS2* and *CALCRL*, which were both upregulated at relapse, on AML aggressiveness and chemotherapy resistance has already been demonstrated [9, 10]. Another candidate gene, *metastasis suppressor 1* (*MTSS1*), was significantly downregulated at relapse.

*MTSS1* is involved in actin filament polymerization, plasma membrane dynamics, receptor internalization, signaling, and transcriptional regulation [11–15]. With few exceptions [16, 17], *MTSS1* acts as a tumor/metastasis suppressor in solid cancers [18–24]. Its expression was reduced in advanced vs. less advanced stages, and low expression correlated with shorter survival in lung, liver, bile duct, and pancreatic cancer [18–22]. In solid tumor cell lines, *MTSS1* reduced proliferation and invasiveness [19, 23, 24]. *Mtss1* knockout mice displayed aberrancies in the B-cell compartment and ultimately developed B-cell lymphomas [25]. Correspondingly, *MTSS1* was downregulated in human B-cell malignancies [25]. *MTSS1* expression was also reduced in diagnostic samples from patients with chronic myeloid leukemia (CML), and restored at remission [26]. Its downregulation was accompanied by increased promoter methylation [26], a feature that was also observed, and associated with parameters related to poor outcome, in pediatric AML and prostate cancer [27, 28]. Ectopic expression of the CML driver oncogene *Bcr-Abl* affected downregulation of *Mtss1*, which was partially counteracted by tyrosine kinase inhibitors [26]. Confirming the tumor-suppressive role of *Mtss1* in CML, its experimental re-expression inhibited colony formation in semi-solid media and decreased leukemic burden in recipient mice [26].

Here, we investigated the role of *MTSS1* in adult AML. Low *MTSS1* expression at diagnosis was associated with poor survival. Correspondingly, experimental downregulation of *MTSS1* in human AML cell lines increased their resistance to araC, DNR, and several other anti-cancer drugs. In an *MLL-AF9*-driven mouse model of AML, knockdown on *Mtss1* drastically shortened time to disease onset, increased leukemic burden, and augmented ex vivo chemotherapy resistance.

## Materials and methods

Additional and more detailed method descriptions as well as information on patient samples and healthy controls are provided in the Supplementary Methods.

### Knockout and knockdown of *MTSS1* in human and murine cells

To achieve knockout or knockdown of *MTSS1*, human AML cell lines were lentivirally transduced with pLenti-Cas9-GFP (Addgene #86145) containing *Cas9* and an sgRNA (sgCtrl, sgMTSS1_1, sgMTSS1_2, sgMTSS1_3, sgMTSS1_4), or with pLKO.1-puro-CMV-TagRFP™ expressing shRNAs (shCtrl, shMTSS1_1, shMTSS1_2; MISSION® shRNA Library; Sigma-Aldrich, St. Louis, MO, USA), respectively. Spleen cells from mice with *MLL-AF9*-driven AML were transduced with pRRL-SFFV-GFP-mirE-PGK-NeoR expressing shRNAs targeting murine *Mtss1* (shMtss1_1, shMtss1_2) or a control shRNA targeting the *Renilla luciferase* gene (shCtrl). sgRNA and shRNA vectors and transduction procedures are described in the Supplementary Methods.

### Drug treatment, and cell viability and apoptosis assays

For viability and AnnexinV assays, human cell lines were incubated with the indicated concentrations of araC, vincristine, or regorafenib (MCE, Monmouth Junction, NJ, USA) for 2 days, or of DNR for 1 day. Murine leukemic cells (LCs) were incubated with the indicated concentrations of DNR or doxorubicin for 2 days. All drugs except regorafenib were provided by the dispensary of the General Hospital, Vienna, Austria. Metabolic activity as a proxy for cell viability was measured using the CellTiter-Glo® Luminescent Cell Viability Assay (Promega, Madison, WI, USA). To quantify the proportions of apoptotic cells after drug treatment, cells were stained with AnnexinV-APC (BD Biosciences, Franklin Lakes, NJ, USA). For the assessment of caspase-3 activation, cells were incubated with araC or DNR for the indicated times, and samples were subjected to immunoblot analysis.

### Drug screen

A library comprising 106 different compounds [29], most of which are approved for oncological indications, and which represent 13 different drug classes (Supplementary Table S1), was screened. TF-1-derived *MTSS1* knockout and control cells were incubated for 2 days with eight different concentrations of each compound, and viability was measured using the CellTiter-Glo® Luminescent Cell Viability Assay. Area-under-the-curve values were compared between knockout and control clones.

### RNA sequencing

RNA sequencing (RNA-seq) was performed on TF-1-derived *MTSS1* knockout and control cells. RNA was isolated from pooled single-cell clones using TRIzol® (ThermoFisher Scientific, Waltham, MA, USA). Sequencing was carried out by the Next Generation Sequencing core facility of the Vienna BioCenter, Vienna, Austria. Reads were mapped to the human genome using HISAT2 v2.1.0. Differentially expressed genes were identified with DESeq2 in R v3.6.1 and visualized using the EnhancedVolcano package in R v4.0.2.

### Congenic mouse model

To characterize the effects of *Mtss1* on leukemogenesis in vivo, a C57BL/6-based, *MLL-AF9*-driven mouse model of AML was employed [9, 30, 31]. In order to moderate disease aggressiveness, the model was based on *MLL-AF9*-transduced common myeloid progenitors [31]. LCs (LC^MLL-AF9^) from the spleens of recipients of pMSCV_*MLL-AF9*_IRES_Venus [32] -transduced common myeloid progenitors were further transduced with shMtss1_1, shMtss1_2, or shCtrl, and transplanted into sub-lethally irradiated female C57BL/6 mice (*n* = 4 per group). Terminally ill mice were sacrificed and peripheral blood, bone marrow (BM), and spleen cells were collected. The number of mice was chosen based on previous studies [9, 10]. No blinding and, since mice were of homogeneous origin, sex, and age, no randomization were applied.

### Statistical analyses

For experiments with cell lines, at least three biological replicates were performed, and data are presented as means ± SEM. For patient samples, data from technical replicates are shown as means ± SD. Student’s two-sided *t*-test was used to assess significances of differences between two independent groups, one-way ANOVA followed by Dunnett’s Multiple Comparison test for comparisons between multiple independent groups and a single control group, and two-way ANOVA followed by Bonferroni’s post-hoc test for multiple groups with ≥2 factors. Tests were performed using GraphPad Prism 6 software (GraphPad Software, San Diego, CA, USA). *p* values <0.05 were considered significant. For experiments with TF-1-derived *MTSS1* knockout cells, all clones were compared to TF-1_sgCtrl_C1. Comparable results were obtained relative to TF-1_sgCtrl_C2.

## Results

### Downregulation of *MTSS1* is associated with relapse and poor outcome in AML

**Fig. 1:**
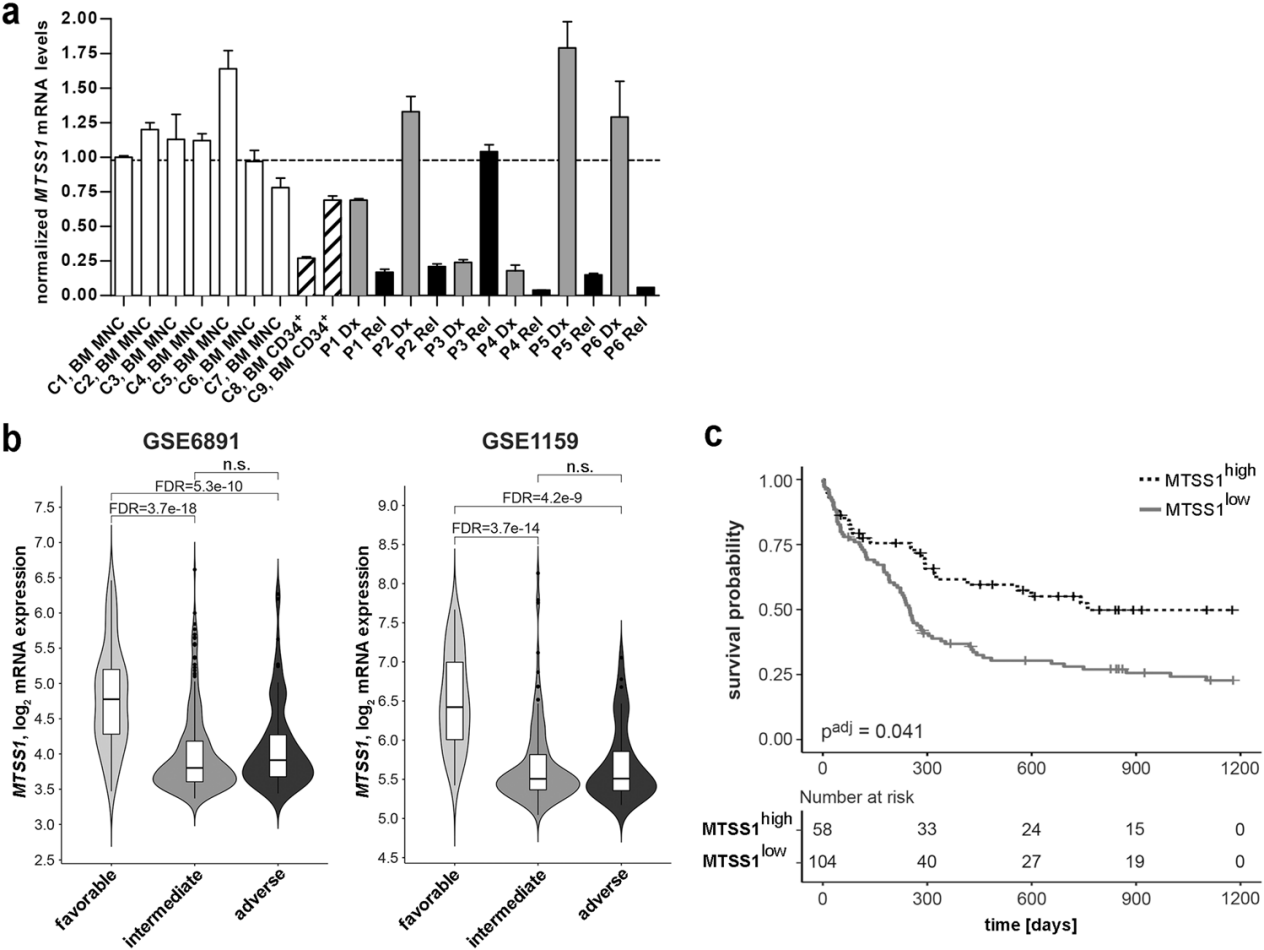
Low *MTSS1* expression is associated with relapse and poor survival in AML. **a***MTSS1* mRNA levels in six paired diagnosis (Dx) - relapse (Rel) mononuclear cell samples from patients with AML (P1-P6) and in healthy controls (C1–C9). BM bone marrow, MNC mononuclear cells, CD34^+^ CD34 positive hematopoietic stem and progenitor cells. *MTSS1* mRNA levels were determined by qRT-PCR and normalized to those of *ß-2-microglobulin* and to sample C1 using the ΔΔC_T_ method. Mean + SD from technical replicates. Dashed line, mean of C1–C9. **b** Left panel: *MTSS1* mRNA levels in the cytogenetically favorable, intermediate, and poor risk groups in AML data set GSE6891. Right panel: *MTSS1* mRNA levels in the ELN favorable, intermediate, and poor risk groups in AML data set GSE1159. FDR false discovery rate after correction for all probe sets present on the arrays, n.s. not significant. **c** Kaplan–Meier curves relating the expression of *MTSS1* (Affymetrix probe set 210360_s_at) to overall survival in AML data set GSE12417 (*n* = 162; age, 17–83 years; cytogenetically normal AML). The cut-off for high vs. low *MTSS1* expression was determined using maximally selected rank statistics. Significance was calculated using the log-rank test; multiplicity correction was performed according to Altman et al. [51].

In a previous genome-wide gene expression analysis on paired samples from patients with AML, we had found that *MTSS1* was significantly downregulated at relapse compared to diagnosis [8]. These results were confirmed by qRT-PCR, using six paired diagnosis-relapse mononuclear cell samples from patients with AML (Fig. 1a). BM mononuclear cells and BM CD34^+^ hematopoietic stem and progenitor cells from seven and two healthy donors, respectively, served as controls (Fig. 1a). *MTSS1* expression was downregulated at relapse in 5/6 samples; the average reduction across all samples was 8.1-fold (Fig. 1a).

To gain further knowledge about the expression of *MTSS1* in AML, samples in the publicly available data sets GSE6891 [33] and GSE1159 [34] were grouped into favorable, intermediate, and poor risk groups according to cytogenetic risk [35] and ELN recommendations [36], respectively. *MTSS1* mRNA levels in the intermediate and poor risk groups were similar, but significantly lower than in the favorable risk groups (Fig. 1b). Furthermore, survival analyses revealed that low *MTSS1* expression correlated significantly with shorter overall survival in two independent patient cohorts (GSE12417 [37], GSE37642 [38]; Fig. 1c and Supplementary Fig. S1). In a third cohort (GSE6891 [33]), a trend (adjusted *p* = 0.057) in the same direction was observed (Supplementary Fig. S1).

In conclusion, these data show that low *MTSS1* expression is associated with chemotherapy resistance and poor outcome in AML.

### The expression of *MTSS1* in AML is regulated through promoter methylation and decreases in response to chemotherapeutic drugs

**Fig. 2:**
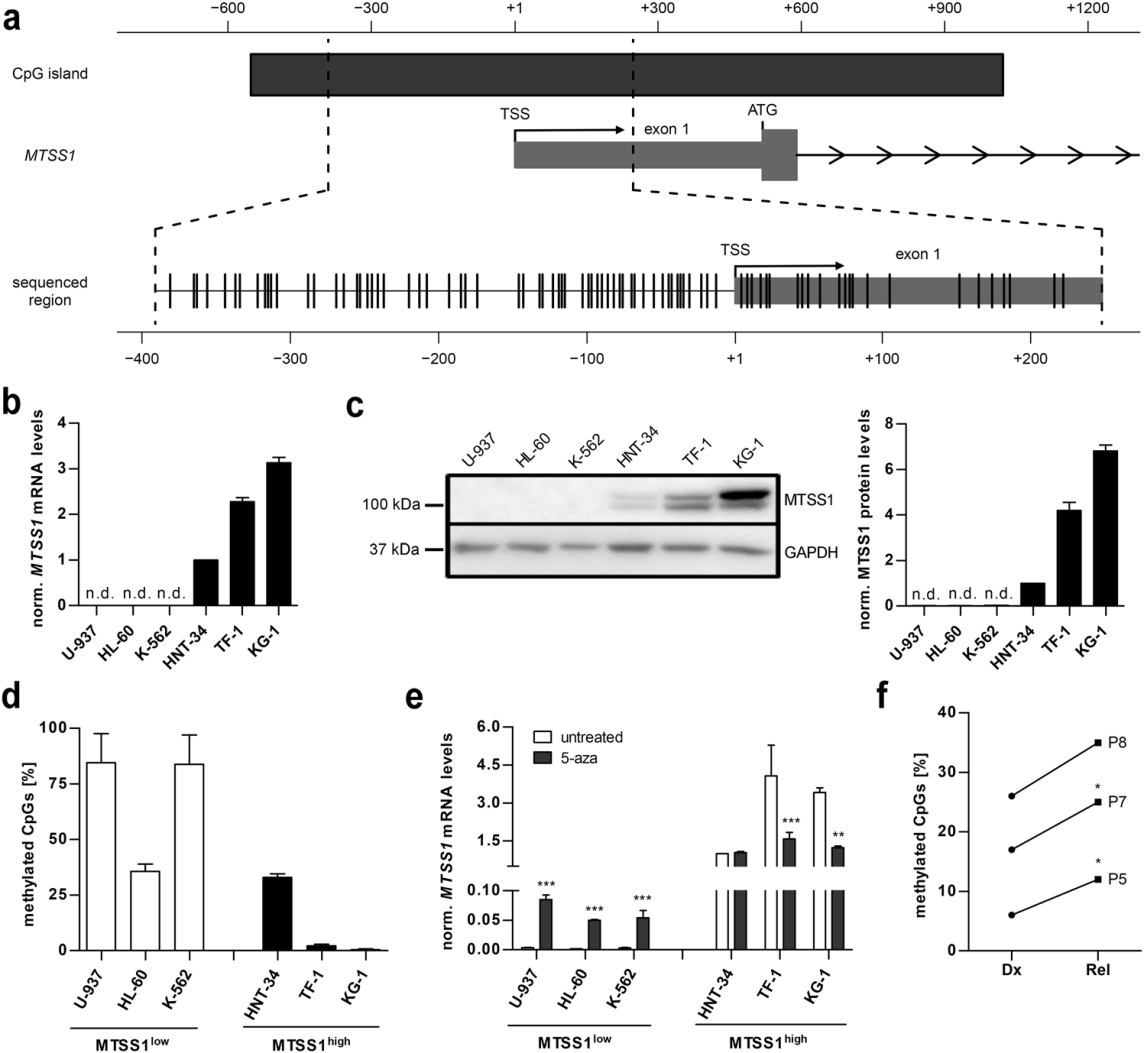
*MTSS1* expression is regulated by promoter methylation in AML. **a** Schematic of the genomic region surrounding the transcription start site (TSS) of *MTSS1* (transcript variant NM_014751). The CpG island is depicted by a dark gray bar; individual CpGs are indicated by vertical lines. ATG, translation start codon. Arrowheads indicate direction of transcription. **b** *MTSS1* mRNA levels in six human myeloid cell lines were determined by qRT-PCR, and normalized to *ß-2-microglobulin* mRNA levels and to HNT-34 cells using the ΔΔC_T_ method. Mean + SEM, *n* = 3. n.d. not detectable. **c** MTSS1 protein levels were determined by immunoblot analysis; GAPDH was used as a loading control. The MTSS1 antibody detects two differently sized proteins derived from different splice variants. Left panel, representative experiment. Right panel, quantification. MTSS1 expression was normalized to GAPDH expression and to HNT-34 cells. Mean + SEM, *n* = 3. n.d. not detectable. **d** The methylation status of a ~650 bp region surrounding the *MTSS1* TSS in six human myeloid cell lines was determined by bisulfite sequencing. White bars, MTSS1^low^ cell lines; black bars, MTSS1^high^ cell lines. Mean + SEM, *n* = 5 cloned PCR products per cell line. **e** Effect of the DNA methyltransferase inhibitor 5-aza-2′-deoxycytidine (5-aza) on *MTSS1* expression. The indicated human myeloid cell lines were treated with 5-aza (black bars) or not (white bars) for 4 days, and *MTSS1* mRNA levels were determined by qRT-PCR as described in (**b**). Mean + SEM, *n* = 3. **p < 0.01, ***p < 0.001; two-way ANOVA followed by Bonferroni’s post-hoc test. **f** Methylation status of a ~650 bp region surrounding the *MTSS1* TSS in three paired diagnosis (Dx) - relapse (Rel) samples from patients with AML. Mean, *n* = 5 cloned PCR products per sample. *p < 0.05, paired two-sided Student’s *t*-test.

Downregulation of *MTSS1* in CML vs. healthy control samples was associated with increased methylation of a ~650 base pair region that surrounds the *MTSS1* transcription start site (Fig. 2a) [26]. We therefore asked whether DNA methylation also contributed to the regulation of *MTSS1* in AML. Among six human malignant myeloid cell lines, HNT-34, TF-1, and KG-1 did, and U-937, HL-60, and K-562 did not express *MTSS1* at both the mRNA and protein levels (Fig. 2b, c). Bisulfite sequencing revealed that fewer than 5% of the 83 analyzed CpGs were methylated in the MTSS1^high^ cell lines KG-1 and TF-1, while over 80`% were methylated in the MTSS1^low^ cell lines U-937 and K-562 (Fig. 2d and Supplementary Fig. S2a). One cell line from each group, HL-60 and HNT-34, showed an intermediate pattern, with methylation present only in the 5′-region of the analyzed sequence (Fig. 2d and Supplementary Fig. S2a). These data suggest that methylation is an important, but not the only, determinant of *MTSS1* expression. Further supporting its role, inhibition of DNA methyltransferases with 5-aza-2′-deoxycytidine resulted in strong upregulation of the *MTSS1* mRNA in U-937, HL-60, and K-562 cells (Fig. 2e), which was accompanied by increased MTSS1 protein expression in U-937 and HL-60 (Supplementary Fig. S2b). Curiously, the opposite effect was observed in the MTSS1^high^ cell lines TF-1 and KG-1: both *MTSS1* mRNA and protein decreased upon 5-aza-2′-deoxycytidine treatment (Fig. 2e and Supplementary Fig. S2b). This unexpected finding may be due to regulation of *MTSS1* by other methylation-sensitive macromolecules (see Discussion).

Genomic DNA from diagnosis and relapse from three patients with AML was subjected to bisulfite sequencing as described above. In accordance with the downregulation of the *MTSS1* mRNA at this disease stage (Fig. 1a), *MTSS1* promoter methylation was increased at relapse (Fig. 2f and Supplementary Fig. S2c).

Next, we asked whether AML chemotherapy could directly affect *MTSS1* expression, and thus potentially contribute to its downregulation at relapse. Exposure of the MTSS1^high^ cell lines HNT-34, TF-1, and KG-1 to IC_50_ concentrations of araC or DNR caused slight to moderate reductions of *MTSS1* mRNA levels, and substantial decreases of the MTSS1 protein (Supplementary Fig. S2d, e).

In summary, these data suggest that the expression of *MTSS1* in AML is regulated on several levels, and promoter methylation plays an important part in its control.

### Experimental downregulation of *MTSS1* augments resistance of human AML cell lines to araC and DNR

To investigate the function of *MTSS1*, a gene knockout was performed in the human AML cell line TF-1 using two sgRNAs, both of which target the first exon of *MTSS1* downstream of the ATG codon (sgMTSS1_1, sgMTSS1_2; Supplementary Fig. S3a). For each sgRNA, two single-cell clones with a full knockout as determined by immunoblot analysis were selected for further experiments (Supplementary Fig. S3b). Single-cell clones from transductions with sgCtrl, targeting the *Renilla luciferase* gene, were used as controls (Supplementary Fig. S3b). At first sight counter-intuitively for a tumor suppressor, elimination of *MTSS1* expression moderately reduced viable cell numbers (Supplementary Fig. S3c), an effect that was not observed in the other experimental models used in this study (see below, and Discussion).

**Fig. 3:**
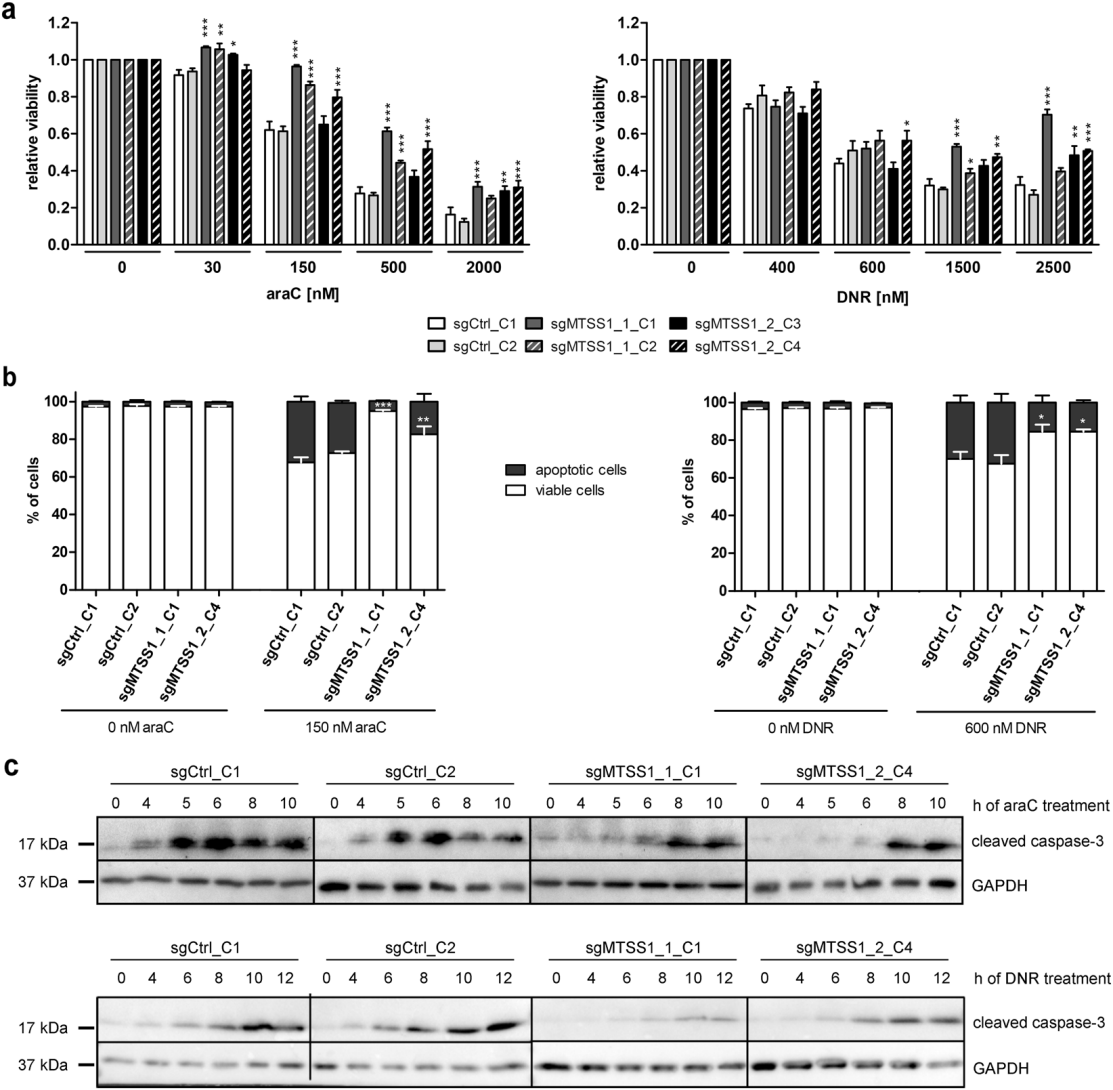
Knockout of *MTSS1* augments araC and DNR resistance of a human AML cell line. **a** TF-1 clones were incubated with the indicated concentrations of araC for 2 days, or of DNR for 1 day, and metabolic activity was determined as a proxy for viability. **b** TF-1 clones were incubated with or without 150 nM araC for 2 days, or with or without 600 nM DNR for 1 day, and stained with AnnexinV. AnnexinV^–^ cells were considered viable, and AnnexinV^+^ cells apoptotic. **a**, **b** Mean + SEM, *n* = 3. *p < 0.05, **p < 0.01, ***p < 0.001, two-way ANOVA followed by Bonferroni’s post-hoc test. **c** TF-1 clones were incubated with 2 µM araC or 1 µM DNR for up to 10 or 12 h, respectively, and cleaved caspase-3 was detected by immunoblot analysis. GAPDH was used as loading control. Representative blots; for quantification, see Supplementary Fig. S3e.

Because the above-described expression patterns of *MTSS1* in primary AML samples suggest a possible role of this gene in chemotherapy responsiveness, *MTSS1*-proficient and -deficient TF-1-derivative cell lines were treated with various concentrations of araC or DNR and subjected to viability assays. The *MTSS1* knockout clones exhibited increased resistance toward araC and DNR compared to the control clones (Fig. 3a). These results were confirmed through AnnexinV assays: knockout of *MTSS1* did not affect the basal rate of apoptosis, but decreased the proportions of apoptotic (AnnexinV^+^) cells formed in response to araC or DNR (Fig. 3b and Supplementary Fig. S3d). Similarly, drug-induced activation of caspase-3, determined via immunoblot analysis, was delayed and decreased in *MTSS1* knockout vs. control clones (Fig. 3c and Supplementary Fig. S3e). To query whether the observed differences in chemotherapy responsiveness were due to differences in drug uptake, we made use of the auto-fluorescent properties of DNR. Flow cytometry after incubation with various concentrations of DNR did not reveal any differences in drug accumulation between knockout and control cells (Supplementary Fig. S4a, b). Next, we asked whether *MTSS1* affected the extent of chemotherapy-induced DNA damage. As expected, the abundance of γH2AX, indicative of DNA double strand breaks and measured by flow cytometry, increased in response to araC and DNR (Supplementary Fig. S5a, b). *MTSS1* knockout clones displayed moderately lower levels of γH2AX compared to control clones after treatment with araC (Supplementary Fig. S5a, b). With DNR, a similar effect became evident only after drug wash-out (Supplementary Fig. S5a, b). These observations suggested that MTSS1 downregulation might increase the cells’ ability to repair chemotherapy-induced DNA damage (Supplementary Fig. S5a, b). Accordingly, the levels of the DNA damage response kinase WEE1 and its target, p-CDK1, were elevated in *MTSS1* knockout vs. control clones (Supplementary Fig. S5c). Thus, knockout of *MTSS1* decreased the sensitivity of AML cells to araC and DNR in a manner involving an increased DNA damage response.

**Fig. 4:**
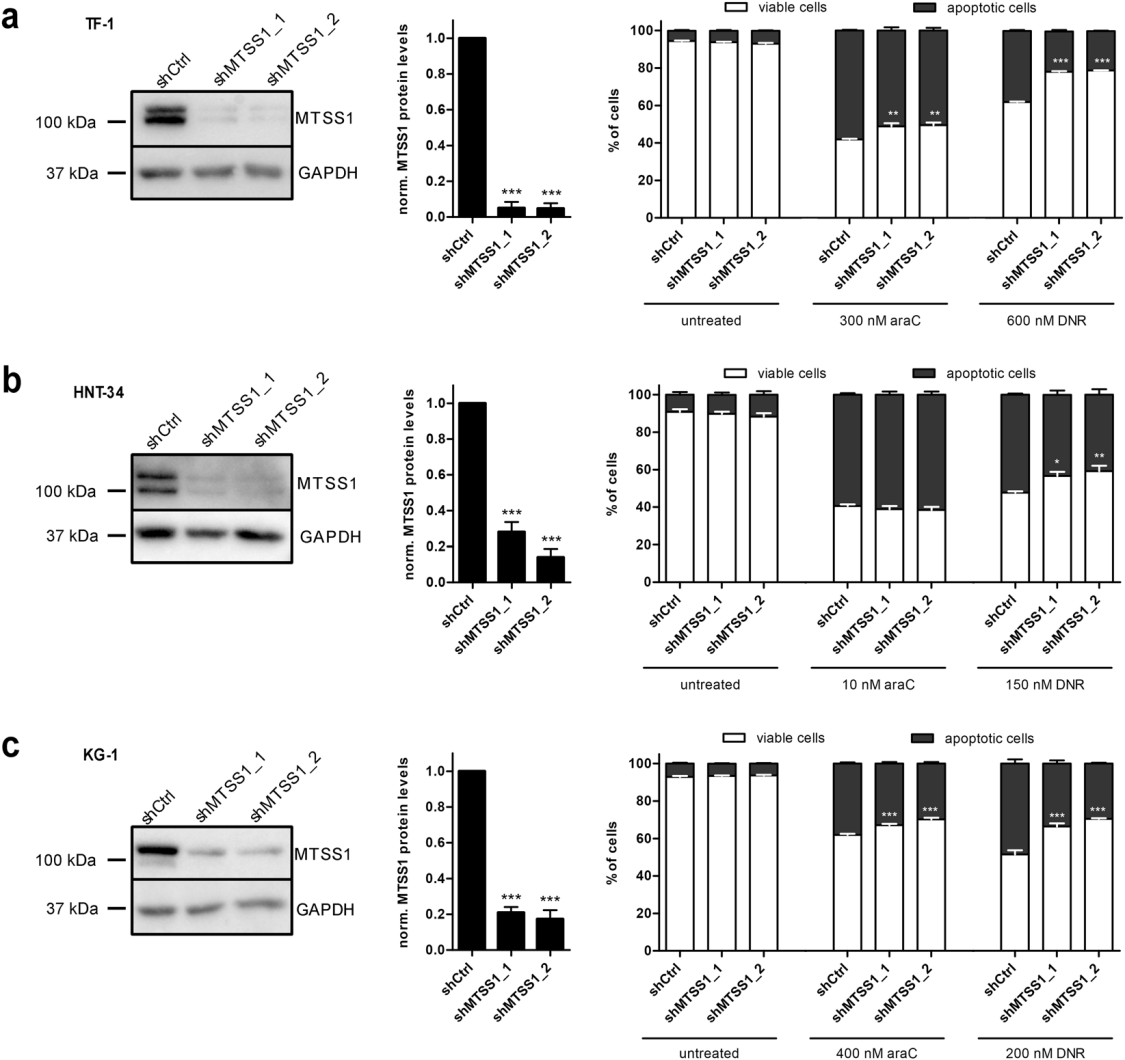
Knockdown of *MTSS1* enhances araC and DNR resistance of human AML cell lines. TF-1 (**a**), HNT-34 (**b**), and KG-1 (**c**) cells were transduced with lentiviral vectors expressing shRNAs targeting MTSS1 (shMTSS1_1, shMTSS1_2) or a non-targeting control shRNA (shCtrl). Downregulation of MTSS1 was validated by immunoblot analysis. Cells were incubated with the indicated concentrations of araC for 2 days or of DNR for 1 day, and stained with AnnexinV. AnnexinV^–^ cells were considered viable, and AnnexinV^+^ cells apoptotic. Left panels, representative immunoblots; middle panels, quantifications of immunoblots; right panels, AnnexinV assays. Mean + SEM, *n* = 3. *p < 0.05, **p < 0.01, ***p < 0.001. Middle panels: Student’s two-sided *t*-test, right panels: two-way ANOVA followed by Bonferroni’s post-hoc test.

To account for the fact that *MTSS1* expression is not lost, but only reduced, at relapse, and to confirm the above-described results in additional cell line models, *MTSS1* was knocked down in AML cell lines TF-1, HNT-34, and KG-1. Two different *MTSS1* shRNAs (shMTSS1_1, shMTSS1_2) or a non-targeting control shRNA (shCtrl) were introduced into each of these cell lines. Efficient knockdown of MTSS1 was confirmed by immunoblot analysis (Fig. 4a–c). In contrast to the CRISPR/Cas9-induced loss of MTSS1, its shRNA-induced reduction did not affect cell proliferation (Supplementary Fig. S6a, c, e). However, like the knockout, the *MTSS1* knockdown increased resistance to araC and DNR as determined through viability and AnnexinV assays (Fig. 4a–c and Supplementary Fig. S6b, d, f).

In summary, these data show that downregulation of *MTSS1* augments the DNA damage response and increases resistance to drugs used in standard AML therapy.

### Downregulation of *MTSS1* increases resistance of AML cells to various drugs approved for oncological indications

**Fig. 5:**
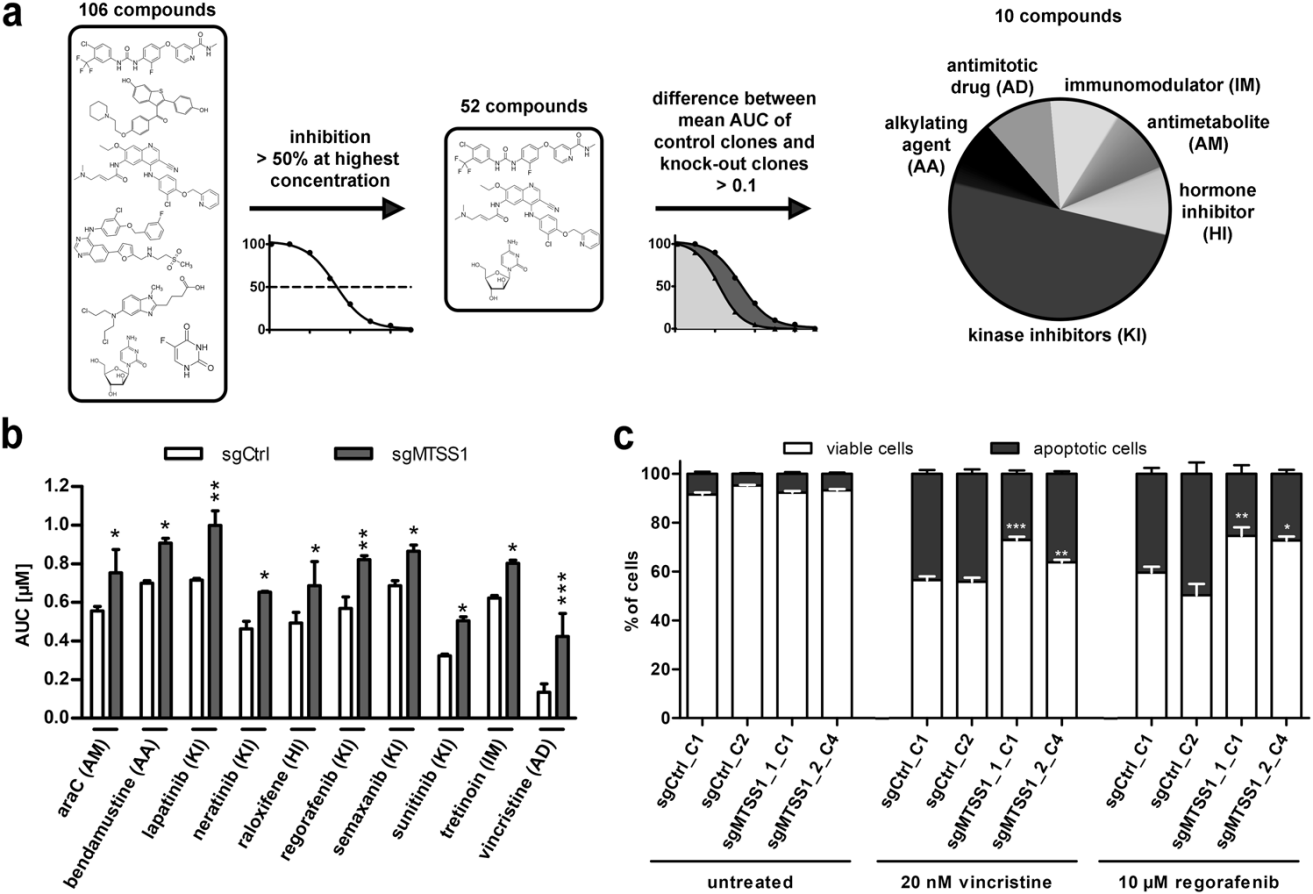
*MTSS1* knockout enhances resistance of an AML cell line to anti-cancer drugs representing various classes. TF-1_sgCtrl_C1, TF-1_sgCtrl_C2, TF-1_sgMTSS1_1_C1, and TF-1_sgMTSS1_2_C4 were subjected to a drug screen based on a library comprising 106 compounds, most of which were approved for oncological indications. **a** Schematic of the drug screen. AUC area-under-the-curve, AA alkylating agent, AD antimitotic drug, AM antimetabolite, IM immunomodulatory substance, HI hormone inhibitor, KI kinase inhibitor. **b** AUC values for the ten drugs with differential activity toward TF-1 control and knockout clones. For drug concentrations, see Supplementary Table S3. Mean + SEM, *n* = 2. **c** TF-1 derivative cell lines were incubated with the indicated concentrations of vincristine or regorafenib for 2 days, and the proportions of apoptotic cells were determined by staining with AnnexinV. Mean + SEM, *n* = 3. (**b**, **c**) *p < 0.05, **p < 0.01, ***p < 0.001, two-way ANOVA followed by Bonferroni’s post-hoc test.

To investigate whether *MTSS1* affected cellular sensitivity to substances other than araC and DNR, a drug library screen was performed on *MTSS1*-proficient and -deficient TF-1 clones (sgCtrl_C1, sgCtrl_C2, sgMTSS1_1_C1, and sgMTTS1_2_C4). The library comprised 106 compounds representing 13 different drug classes [29]; 87 of the drugs were approved for oncological indications. Fifty-two drugs reduced the mean viability of the control clones by at least 50% at the highest concentration. Using a difference of 0.1 between the mean area-under-the-curve values for the *MTSS1* knockout and the control clones as threshold, ten drugs from various classes were found to exhibit altered—and in fact, in all cases reduced—toxicity toward the knockout clones (Fig. 5a, b). This list included the antimitotic drug vincristine, the tyrosine kinase inhibitor regorafenib, and, validating the screening results, the antimetabolite araC (Fig. 5b and Supplementary Table S1). Regorafenib is being tested in a phase I study in patients with AML (NCT03042689), and vincristine is approved for AML; these drugs were therefore selected for validation. Indeed, viability and AnnexinV assays on TF-1 knockout and control clones confirmed the decreased sensitivity of *MTSS1*-deficient cells to both compounds (Fig. 5c and Supplementary Fig. S7a, b).

In summary, downregulation of *MTSS1* increases the resistance of AML cells not only to standard AML therapeutics, but to members of several different classes of anti-cancer drugs.

### Knockout of *MTSS1* alters the expression of myeloid transcription factor target genes

**Fig. 6:**
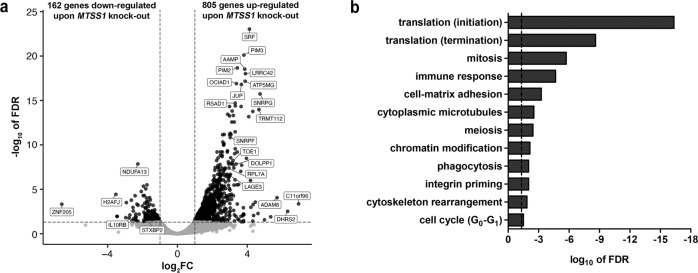
Knockout of *MTSS1* in a human AML cell line leads to deregulation of numerous genes. TF-1 derivative cell lines with an sgRNA-mediated knockout of *MTSS1* and corresponding controls were subjected to RNA-seq. **a** Volcano plot showing the log_2_-fold change (log_2_FC) between knockout and control cells vs. the –log_10_-transformed false discovery rate (FDR) for each gene. Dashed horizontal line, significance level (FDR = 0.05), dashed vertical lines, log_2_FC cutoffs (−1 and 1, respectively). Genes with an FDR > or <0.05 are represented by gray and black dots, respectively. Names of exemplary genes are given, for full list of differentially expressed genes, see Supplementary Table S2A. **b** Cellular processes regulated by *MTSS1* (GeneGo MetaCore).

To gain insight into the molecular changes triggered by loss of *MTSS1* expression, its impact on genome-wide gene expression patterns of TF-1 cells was assessed. To this end, pools of four single-cell clones for each of sgMTSS1_1, sgMTSS1_2, sgMTSS1_3, sgMTSS1_4, and three such pools for sgCtrl were used. Successful gene knockout in sgMTSS1 clones was verified by immunoblot analysis (Supplementary Fig. S8a), and the effects of sgMTSS1_3 and sgMTSS1_4 on the chemotherapy responsiveness of TF-1 cells were shown to be similar to those of sgMTSS1_1 and sgMTSS1_2 (Supplementary Fig. S8b). RNA-seq revealed 967 genes that were differentially expressed between *MTSS1* knockout and control cells at a false discovery rate <0.05 and a log_2_-fold change >1 or <−1; 805 of these were up-, and 162 downregulated upon loss of MTSS1 (Fig. 6a and Supplementary Table S2A). Gene ontology analysis showed that genes associated with translation, cell cycle, immune response, cell adhesion, cytoskeleton, and transcription were significantly enriched among the *MTSS1* target genes (Fig. 6b and Supplementary Table S2B). Further functional annotation revealed an enrichment for genes previously described as responsive to or regulated by transcription factors involved in normal and/or malignant myelopoiesis, e.g., MYC, RUNX1, CREB1, GATA2, STAT3, MEIS1, CEBPA, SPI1 (PU.1), and MECOM [39, 40] (Supplementary Table S2C). The latter finding was confirmed in a list of 73 genes that were deregulated upon loss of *MTSS1* in TF-1 cells, and additionally were correlated with *MTSS1* expression in primary human AML samples (Supplementary Table S2D, E).

In summary, knockout of *MTSS1* affected the expression of a large number of genes, many of which are known targets of key myeloid transcription factors.

### Knockdown of *Mtss1* in a mouse model of AML increases disease aggressiveness and ex vivo drug resistance

**Fig. 7:**
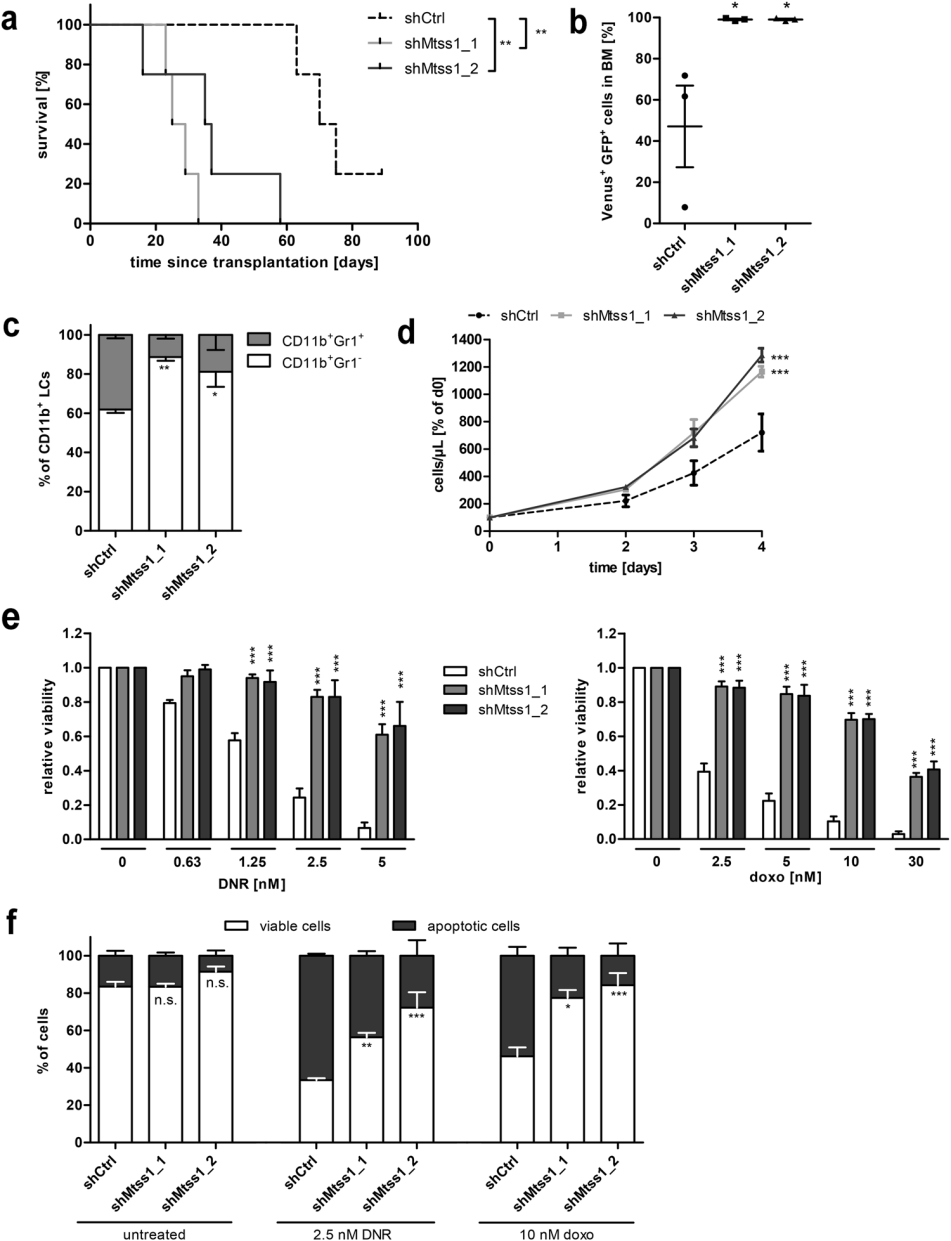
Knockdown of *Mtss1* in *MLL-AF9*-driven murine AML augments disease aggressiveness and ex vivo chemotherapy resistance. **a** Kaplan–Meier plot of mice transplanted with shCtrl or shMtss1 transduced LC^MLL-AF9^ (500 000 Venus^+^ GFP^+^ cells/mouse, *n* = 4 mice/group). One mouse in the control group was still alive at the time of submission. **b**–**f** Analysis of LC^MLL-AF9_shMtss1^ and LC^MLL-AF9_shCtrl^ from BM of terminally ill mice. *n* = 3; mean ± SEM. **b** Leukemic burden in BM, defined as the percentage of Venus^+^ GFP^+^ cells among all viable cells. **c** Myeloid differentiation, determined as the proportion of Gr1^+^ cells among myeloid leukemic (CD11b^+^ Venus^+^ GFP^+^) cells. **d** Cells were seeded at equal densities, and metabolic activity as a proxy for viable cell numbers was determined daily for 4 days. **e** LC^MLL-AF9_shMtss1^ and LC^MLL-AF9_shCtrl^ were incubated with the indicated concentrations of DNR or doxorubicin (doxo) for 2 days, and metabolic activity was determined as a proxy for viability. **f** LC^MLL-AF9_shMtss1^ and LC^MLL-AF9_shCtrl^ were incubated with or without 2.5 nM DNR or 10 nM doxorubicin for 2 days and stained with AnnexinV. AnnexinV^–^ cells were considered viable, and AnnexinV^+^ cells apoptotic. **a**–**f** n.s. not significant, *p < 0.05, **p < 0.01, ***p < 0.001. **a** Log-rank test. **b** One-way ANOVA followed by Bonferroni’s post-hoc test. **c**–**f** Two-way ANOVA followed by Bonferroni’s post-hoc test.

To investigate the impact of *Mtss1* downregulation on AML in vivo, a well-characterized, *MLL-AF9*-driven mouse model of AML was used [30, 31, 41]. The *Mtss1* mRNA was expressed in LC^MLL-AF9^ isolated from BM of mice that had been transplanted with *MLL-AF9*-transduced common myeloid progenitors (Supplementary Fig. S9a). Spleen LC^MLL-AF9^ were transduced with two different shRNAs against *Mtss1* or with a control shRNA, sorted for fluorescence marker (Venus and GFP) positivity, and transplanted into recipient mice. Knockdown of *Mtss1* (confirmed by qRT-PCR in transduced LC^MLL-AF9^; Supplementary Fig. S9b) strongly decreased disease latency (median survival, 72.5, 27, and 36 days for shCtrl, shMtss1_1, and shMtss1_2, respectively; *p* < 0.01 for both shRNAs; Fig. 7a). *Mtss1* knockdown did not significantly affect spleen weight, white and red blood cell count, or platelet count (Supplementary Fig. S9c, d), but strongly increased leukemic burden in terminally ill mice as determined by the proportion of Venus^+^ GFP^+^ cells in their BM (Fig. 7b and Supplementary Fig. S10a). Furthermore, it increased the proportion of immature (Gr1^–^) cells in the myeloid leukemic (CD11b^+^ Venus^+^ GFP^+^) compartment (Fig. 7c and Supplementary Fig. S10b). Consistent with their in vivo properties, LC^MLL-AF9^ containing shMtss1 (LC^MLL-AF9_shMtss1^) also proliferated faster than their shCtrl containing counterparts (LC^MLL-AF9_shCtrl^) ex vivo (Fig. 7d). Finally, LC^MLL-AF9_shMtss1^ isolated from terminally ill mice were substantially more resistant to DNR and to doxorubicin (usually used in mice due to the toxicity of intraperitoneally administered DNR [42]) than LC^MLL-AF9_shCtrl^ (Fig. 7e, f and Supplementary Fig. S10c).

In summary, knockdown of *Mtss1* in an *MLL-AF9*-driven mouse model of AML strongly increased disease aggressiveness and the resistance of LCs to chemotherapeutic drugs.

## Discussion

The multifunctional protein MTSS1 acts as a tumor and/or metastasis suppressor whose downregulation was associated with a poor prognosis in several solid tumor entities [18–22]. *Mtss1* knockout mice, however, developed B-cell lymphomas, pointing toward an anti-oncogenic role of this gene also in the hematopoietic system. *MTSS1* was downregulated in CML, and its experimental over-expression in CML model systems impaired clonogenic growth and tumorigenesis [26]. In AML, *MTSS1* was reported to constitute part of a prognostically relevant, TET2-related 4-gene methylation signature [43]. *MTSS1* mRNA levels were high in AML with prognostically favorable CBF-rearrangements, and low in AML carrying *FLT3*-ITD mutations or a *PML-RARA* fusion, both of which are associated with a poor prognosis under araC/anthracycline based therapy [44, 45]. Furthermore, low *MTSS1* expression was associated with an increased risk of relapse in pediatric AML [27], and with poor overall survival in a cohort of adult patients with cytogenetically normal AML [44].

Here, we present additional evidence for the prognostic relevance of *MTSS1* in AML: *MTSS1* was downregulated at relapse of AML, and in genetically defined intermediate and poor risk subgroups as compared to the corresponding good risk groups. Furthermore, low *MTSS1* mRNA levels were associated with shorter overall survival in three independent patient cohorts. However, the latter finding could not be confirmed in the validation cohorts of GSE12417 and GSE6891. Remarkably, the majority of studies on the prognostic significance of MTSS1 in solid tumors were performed using immunohistochemistry. This raises the possibility that associations between *MTSS1* expression and outcome do not always manifest on the mRNA level, but may be more reliably reflected on the protein level. In fact, *MTSS1* expression appears to be regulated on several levels. Our data show that, like in prostate cancer and CML [26, 28], promoter methylation contributes to the regulation of *MTSS1* in primary AML samples and AML cell lines. However, HL-60 and HNT-34 cells displayed comparable, intermediate *MTSS1* methylation levels despite divergent expression levels, indicating that transcriptional regulation of *MTSS1* in AML includes, but is not restricted to, promoter methylation. Accordingly, *MTSS1* is a confirmed target for several transcription factors in myeloid cells, such as KAISO and REST [26]. Peculiarly, the DNA methyltransferase inhibitor 5-aza-2′-deoxycytidine not only led to the expected upregulation of *MTSS1* in cell lines with promoter methylation, but had the opposite effect in the *MTSS1* unmethylated, MTSS1^high^ cell lines TF-1 and KG-1. This could be due to the existence of a methylation-sensitive antisense transcript, and indeed, the 3′-end of *MTSS1* overlaps with the 3′-end of one of the transcript variants of the *NDUFB9* gene, whose promoter also harbors a CpG island (UCSC genome browser, hg38). Alternatively, methylation-sensitive microRNAs and/or transcription factors could mediate the downregulation of *MTSS1* in response to 5-aza-2′-deoxycytidine treatment. Underscoring the complexity of the regulation of *Mtss1*, the levels of its mRNA were reduced upon expression of *bcr-abl* in murine CML models, but only partially restored by tyrosine kinase inhibitor treatment [26]. Furthermore, even though araC and DNR caused a moderate reduction of *MTSS1* mRNA levels, the downregulation on the protein level was much stronger and more significant. This indicates that chemotherapy is unlikely to directly cause downregulation of the *MTSS1* mRNA at relapse of AML, and points toward a post-transcriptional layer of *MTSS1* regulation. In line with this, *MTSS1* was shown to be a target of several miRNAs [24, 46].

Experimental downregulation of *MTSS1* in three different human AML-derived cell lines and in primary murine AML cells increased their resistance to the standard chemotherapeutics used in AML treatment. This effect was independent of *TP53* status, because it was observed both in cells with wild-type (HNT-34, Supplementary Fig. S11; LC^MLL-AF9^ [47]) and with homozygously mutated (TF-1, KG-1 [48]; Supplementary Fig. S11) *TP53*. Rather, knockdown of *MTSS1* in TF-1 cells increased the levels of WEE1 and p-CDK1, suggesting that increased activity of the S and/or G_2_/M checkpoints may contribute to the increased resistance of MTSS1^low^ cells to araC and anthracyclines. Knockout of *MTSS1* enhanced the activity of Rac1 [14], which in turn was related to increased WEE1 levels and G_2_/M checkpoint activity [49, 50], suggesting a possible molecular link between MTSS1 and WEE1. However, activation of DNA damage checkpoints is unlikely to be the only mechanism through which *MTSS1* downregulation confers therapy refractoriness, because reduced MTSS1 levels augmented resistance not only to classical DNA-damaging AML therapeutics, but also to several other approved anti-cancer agents representing various drug classes. *MTSS1* may thus play a wider role in the response to drugs with the potential to eliminate malignant cells, which may be of relevance not only for the application of targeted therapy in AML, but also for other tumor entities in which downregulation of *MTSS1* plays a role [18–22, 44].

In addition to its effects on chemotherapy responsiveness, *MTSS1* affected the proliferation of AML cells. Most impressively, its knockdown greatly accelerated leukemia development in an *MLL-AF9*-driven mouse model of AML. *MLL-AF9* can transform both hematopoietic stem and progenitor cells, giving rise to very aggressive or a somewhat more slowly progressing AML-like disease, respectively, upon transplantation into congenic recipient mice [30, 31, 41]. We based our model on *MLL-AF9*-transduced common myeloid progenitors in order to facilitate detection of a disease-accelerating effect of the *Mtss1* knockdown in vivo. Consistent with the in vivo phenotype, LC^MLL-AF9_shMtss1^ also proliferated faster than LC^MLL-AF9_shCtrl^ ex vivo, yet their basal rates of apoptosis were comparable, similar to our observations in the human *MTSS1* knockdown or knockout AML cell lines. Other than with the mouse cells, however, the knockdown also did not alter the proliferation of the cell lines. CRISPR/Cas9-mediated elimination of *MTSS1* in TF-1 cells even retarded their proliferation, possibly indicating a requirement for minimal levels of the multifunctional MTSS1 protein to maintain optimal cell division rates.

In summary, our data show that downregulation of *MTSS1* is associated with relapse and shortened survival in AML. It promotes leukemogenesis in an AML mouse model in vivo, and augments resistance to drugs used in standard AML therapy, as well as to a variety of other anti-cancer therapeutics. These results suggest reduced *MTSS1* expression as a novel factor contributing to disease aggressiveness, therapy refractoriness, and poor outcome in AML.

## Supplementary information

Supplementary Methods and Material

Supplementary Table S1

Supplementary Table S2A

Supplementary Table S2B

Supplementary Table S2C

Supplementary Table S2D

Supplementary Table S2E
