# Supplementary material for: Downregulation of MTSS1 in acute myeloid leukemia is associated with a poor prognosis, chemotherapy resistance, and disease aggressiveness
Source: Leukemia. 2021 Mar 29;35(10):2827–39. doi: 10.1038/s41375-021-01224-2 (PMC8478650; doi:10.1038/s41375-021-01224-2)
Supplement: Supplementary file 1 — Supplementary Methods and Material [file 41375_2021_1224_MOESM1_ESM.doc]

Supplementary information for manuscript:

**Down-regulation of *MTSS1* in acute myeloid leukemia is associated with a poor prognosis, chemotherapy resistance, and disease aggressiveness**

**This supplementary information includes:**

1. Supplementary methods
2. Supplementary Table S1 (caption only)
3. Supplementary Table S2 (caption only)
4. Supplementary Table S3
5. Supplementary Table S4
6. Supplementary Table S5
7. Supplementary Figure Legends
8. Supplementary Figures S1 to S11

**Supplementary Methods**

**Ethics approval and consent to participate**

Experiments with samples from patients with AML, who had given informed consent to study participation, were approved by the Ethics Committee of the Medical University of Vienna (EK179/2011).

Animal experiments were approved by the Animal Ethics Committee of the Medical University of Vienna and the Austrian Federal Ministry of Education, Science, and Research (BMWFW-66.009/0309-WF/V/3b/2015, issued on 3 Nov 2015). Austrian and Federation of European Laboratory Animal Science Associations guidelines to minimize animal distress and suffering were followed.

**Patient samples and healthy controls**

Expression of *MTSS1* was analyzed in Ficoll-enriched mononuclear cells from six paired diagnosis-relapse samples from patients with AML, which had also been used previously for genome-wide gene expression analysis [1]. Genomic DNA from one of these (P5), and from two additional pairs was used for promoter methylation analysis. Samples were collected at the Medical University of Graz, Austria. Patients’ clinical characteristics are summarized in Supplementary Table S3. Bone marrow (BM) mononuclear cells (Lonza, Basel, Switzerland) and CD34^+^ hematopoietic stem and progenitor cells (Lonza) from seven and two healthy donors, respectively, served as controls.

**Cell culture of human and murine cells**

The human malignant myeloid cell lines HL-60 (source: P. Valent, Medical University of Vienna, Austria), U-937 (source: C. Fonatsch, Medical University of Vienna, Austria), K-562 (source: C. Fonatsch), HNT-34 (source: K. Morishita, Musashino Red Cross Hospital, Tokyo, Japan), KG-1 (source: Deutsche Sammlung von Mikroorganismen und Zellkulturen GmbH, Braunschweig, Germany), and TF-1 (source: M. Odero, University of Navarra, Pamplona, Spain) were cultured in RPMI 1640 (Thermo Fisher Scientific, Waltham, MA, USA) containing 10% fetal bovine serum (FBS, Thermo Fisher Scientific), 1x penicillin-streptomycin (Sigma-Aldrich, St. Louis, MO, USA), and, in the case of TF-1, 2 ng/mL recombinant human GM-CSF (Peprotech, Hamburg, Germany). Phoenix GP cells (source: H. Stockinger, Medical University of Vienna, Austria) were grown in DMEM (Thermo Fisher Scientific) supplemented with 10% FBS and 1x penicillin-streptomycin-glutamine (Thermo Fisher Scientific). All cell lines were tested regularly for *Mycoplasma* contamination using the MycoAlert™ Mycoplasma Detection Kit (Lonza). Leukemic cells from spleen or BM of mice with *MLL-AF9* driven AML were grown in IMDM (Thermo Fisher Scientific) supplemented with 10% FBS, 1x penicillin-streptomycin, 2 mM L-glutamine (Thermo Fisher Scientific), 10 ng/mL mIL-6 (BioLegend, San Diego, CA, USA), 10 ng/mL mIL-3, 10 ng/mL mFlt3L, 10 ng/mL mTPO, and 25 ng/mL mSCF (all from Peprotech). Logarithmically growing cells were used for all experiments.

**sgRNA and shRNA constructs**

For the generation of *MTSS1* knock-out clones, four single guide RNAs (sgRNAs) specific for the human *MTSS1* gene and one control sgRNA targeting the *renilla luciferase* gene were designed using the web tool CHOPCHOP (http://chopchop.cbu.uib.no/) [2, 3]. sgRNA constructs were prepared as described by Ran *et al.* 2013 [4]. Briefly, forward and reverse DNA oligonucleotides including vector specific overhangs (Eurofins Genomics, Luxembourg, Luxembourg; Supplementary Table S4) were phosphorylated by incubation with T4 polynucleotide kinase (NEB, Ipswich, MA, USA) for 30 min at 37 °C and annealed in a thermocycler by gradually cooling from 95 °C to 25 °C (5 °C/min). Annealed oligonucleotides were ligated into the BsmBI site of the recipient vector pLenti-Cas9-GFP (Addgene #86145).

Two different shRNAs targeting the 3’-UTR of the human *MTSS1* gene and a control shRNA, all in pLKO.1-puro-CMV-TagRFP™, were purchased from Sigma-Aldrich (MISSION® shRNA Library; Supplementary Table S4).

Sequences of shRNAs targeting murine *Mtss1* (shMtss1_1 and shMtss1_2) were obtained from [5]. A control shRNA (shRen.713, Supplementary Table S4) in pRRL-SFFV-GFP-mirE-PGK-NeoR was kindly provided by Dr. Johannes Zuber, IMP, Vienna, Austria. To construct shMtss1_1 and shMtss1_2 expression vectors, XhoI and EcoRI restriction sites were added to the respective 97-mer oligonucleotides (Eurofins Genomics; Supplementary Table S4) by PCR with primers 5'miR-Xho1 and 3'miR-EcoR1 (Supplementary Table S4). The resulting products were cloned into pRRL-SFFV-GFP-mirE-PGK-NeoR using standard technologies.

**Lentiviral transduction**

Phoenix GP cells were transfected with sgRNA or shRNA expressing lentiviral vectors along with helper plasmids pMD2.G and psPAX2 using a standard calcium phosphate protocol. After 48 h, virus-containing supernatant was collected and filtered through a 0.45 µm filter. Cell lines were transduced by incubation with lentiviral particles in the presence of 5 µg/mL polybrene (Sigma-Aldrich). In the case of *MLL-AF9-*transformed primary murine AML cells (LC^MLL-AF9^), transductions were performed by centrifugation with polybrene-supplemented lentiviral particles for 45 min at 1 300 rpm and 35 °C. Infection cycles were repeated after 24 and 48 h. Three days after the last cycle, cells were sorted for fluorescence marker positivity (GFP for human sgRNA and murine shRNA constructs, RFP for human shRNA constructs, and Venus for the *MLL-AF9* expression vector) on a BD FACSAria™ Fusion cell sorter (BD Biosciences, Franklin Lakes, NJ, USA). To generate single cell clones of sgRNA-transduced TF-1 cells, they were seeded at 0.3 cells/well, and raised in the presence of 50% TF-1-conditioned medium. These clones and bulk populations of shRNA-transduced TF-1, HNT-34, and KG-1 cells were tested for MTSS1 expression by immunoblot analysis. The MTSS1 antibody (Supplementary Table S5) recognizes the sequence around Val650, encoded by the 3'-most exon, which is shared between all transcript variants. Transduced LC^MLL-AF9^ were tested for *Mtss1* expression by qRT-PCR.

The identities of the mutations induced by Cas9 were determined for TF-1_sgMTSS1_1_C1 and TF-1_sgMTSS1_2_C4. A region including the target sites of both sgRNAs was amplified from genomic DNA using primers *MTSS1*_exon1_fwd and *MTSS1*_exon_1_rev (Supplementary Table S4). PCR products were cloned and sequenced as described in the section “Preparation of genomic DNA, bisulfite sequencing, and 5-aza-2'-deoxycytidine treatment”. Heterozygous insertions were found in TF-1_sgMTSS1_1_C1 and a homozygous deletion in TF-1_sgMTSS1_2_C4 (Supplementary Fig. S3a).

**Immunoblot analysis**

Protein extracts were prepared using RIPA buffer (50 mM Tris/HCl pH 8.0, 0.1% SDS, 0.5% sodium desoxycholate (all from Sigma-Aldrich), 150 mM NaCl (Carl Roth, Karlsruhe, Germany), 1% Triton X-100 (Roche, Penzberg, Germany)). Protein concentrations were determined using the Bradford assay (Bio-Rad Laboratories, Hercules, CA, USA), and samples were diluted to comparable concentrations with 1x Roti®-Load 1 (Carl Roth). SDS-PAGE was performed using standard methods. The Pierce™ Power Blotter (Thermo Fisher Scientific) was used for semi-dry protein transfer to PVDF membranes (Pall, Port Washington, NY, USA). Alternatively, tank transfer was carried out using the Mini Trans-Blot® cell (Bio-Rad). Membranes were blocked for 1 h at room temperature with 5% non-fat dry milk (AppliChem, Darmstadt, Germany)/TBS-T (40 mM Tris/HCl pH 7.6, 273 mM NaCl, 0.1% Tween 20 (Sigma-Aldrich)) or with 5% bovine serum albumin (BSA, Sigma-Aldrich)/TBS-T. Antibody incubations were also performed in 5% non-fat dry milk/TBS-T or 5% BSA/TBS-T (overnight at 4 °C for primary antibodies, 1 h at room temperature for secondary antibodies). All antibodies are listed in Supplementary Table S5. Immunoblots were developed with SuperSignal West Femto or Pico Chemiluminescent Substrates (Thermo Fisher Scientific), and signals were detected and quantified on a ChemiDoc Touch Imaging System (Bio-Rad).

**Quantitative real-time reverse transcriptase PCR**

Total RNA was extracted using TRIzol® reagent (Thermo Fisher Scientific). cDNA was synthesized with random hexamer primers and M-MLV reverse transcriptase (both from Thermo Fisher Scientific). Quantitative real-time PCR (qRT-PCR) was performed on a StepOnePlus Real-Time PCR System (Thermo Fisher Scientific). For human cell lines, gene-specific TaqMan probes (*MTSS1*: Hs00207341_m1, *β-2-microglobulin*: Hs99999907_m1) and the TaqMan Gene expression Mastermix (all from Thermo Fisher Scientific) were used. For murine samples, gene specific primers (synthesized by Eurofins Genomics; Supplementary Table S4) and the GoTaq® qPCR Master Mix (Promega, Madison, WI, USA) were used. *MTSS1* mRNA levels were normalized to those of *ß-2-microglobulin* and to a reference sample using the ΔΔC_T_ method [6].

**DNR uptake and generation of DNA double strand breaks**

To determine the extent of DNR uptake, TF-1_sgMTSS1_1_C1, TF-1_sgMTSS1_2_C4, TF-1_sgCtrl_C1, and TF-1_sgCtrl_C2 cells were seeded at 100/µL, incubated with 400 or 2 500 nM DNR for 4, 6, or 10 h, and washed once with PBS (Thermo Fisher Scientific). DNR uptake was quantified by flow cytometry (LSR Fortessa, BD Biosciences; PerCP channel).

Drug induced DNA damage was assessed *via* the DNA double strand break binding histone variant γH2AX. TF-1 cells with or without knock-out of *MTSS1* were seeded at a density of 100/µL, incubated with 150 nM araC for 24 h or with 600 nM DNR for 16 h, washed twice with PBS, and incubated in growth media for another 5 h. Untreated controls were kept in parallel cultures for 24 or 16 h, respectively. Cells were harvested after treatment and after the recovery period. They were fixed in 4% formaldehyde (Sigma-Aldrich)/PBS for 15 min at room temperature, permeabilized in 90% methanol (Fisher Scientific, Schwerte, Germany)/PBS for 30 min on ice, washed with PBS, and stained with *γ*H2AX or isotype control antibody (Supplementary Table S5) in 0.5% BSA/PBS for 1 h at room temperature. Flow cytometric analysis was performed on an LSR Fortessa (BD Biosciences).

**Preparation of genomic DNA, bisulfite sequencing, and 5-aza-2'-deoxycytidine treatment**

To analyze the methylation status of the *MTSS1* promoter, 8 × 10^6^ logarithmically growing U-937, HL-60, K-562, HNT-34, TF-1, and KG-1 cells were collected, washed with PBS, and lysed in 300 µL lysis buffer (100 mM NaCl, 10 mM Tris/HCl pH 8.0, 25 mM EDTA pH 8.0 (Sigma-Aldrich), 0.5% SDS, 0.1 mg/mL proteinase K (Thermo Fisher Scientific)) overnight at 55 °C. Proteins were removed by addition of 200 µL 6 M NaCl and centrifugation. Genomic DNA (gDNA) was precipitated from the supernatant by adding 2 volumes of 100% ethanol (Merck, Darmstadt, Germany), collected by centrifugation, dried at 37 °C, and dissolved in Tris-EDTA buffer (Sigma-Aldrich). Bisulfite conversion of gDNA from cell lines and of archival gDNA samples from patients with AML was performed using the EpiTect Bisulfite Kit (QIAGEN, Venlo, Netherlands) according to the manufacturer’s instructions.

A previously reported [7] ~650 bp region of the *MTSS1* CpG island (-381 to +222 relative to the transcriptional start site) was amplified using HotStartTaq polymerase (QIAGEN) and primers MTSS1_*bis*_fwd and MTSS1_*bis*_rev (Supplementary Table S4). To prepare a TA-cloning vector, pBluescript II KS+ was digested with EcoRV (Thermo Fisher Scientific) and purified from an agarose gel using the QIAquick Gel Extraction Kit (QIAGEN). T-overhangs were added by incubation of 5 µg purified vector with 50 U/mL *Taq* polymerase (NEB) and 2 mM dTTP (Thermo Fisher Scientific) in 100 µL 1x Standard *Taq* buffer (NEB) for 2.5 h at 68 °C. The vector was purified with the QIAquick PCR Purification Kit (QIAGEN), and ligated to the agarose gel purified PCR products using the Quick Ligation™ Kit (NEB). Ligations were transformed into NEB-10*β* competent bacteria (NEB). Plasmid DNA was isolated using a standard miniprep protocol, and 5 to 6 PCR clones were submitted to Eurofins Genomics for Sanger sequencing. Methylated CpG residues were identified using the QUMA online tool [8].

To assess the effect of the demethylating agent 5-aza-2'-deoxycytidine (5-aza) on *MTSS1* expression, cells were seeded at 500/µL (HL-60, U-937, HNT-34, KG-1) or at 300/µL (K-562, TF-1), and treated with 5 µM freshly dissolved 5-aza (Sigma-Aldrich; 10 mM stock in RPMI 1640) every day for 4 consecutive days. RNA and protein was isolated from treated cells, and subjected to qRT-PCR and immunoblot analysis as described above.

**Cell proliferation assays**

To compare the proliferation of cells with or without experimental down-regulation of *MTSS1*, the respective cell lines or primary murine BM cells were seeded at a density of 100 cells/µL in clear 96-well-plates (TPP, Trasadingen, Switzerland) and incubated at 37 °C for up to 4 days. On each day, resazurin (Sigma-Aldrich, 550 µM stock in RPMI 1640) was added to a subset of wells to a final concentration of 55 µM. After 6 h at 37 °C, fluorescence was measured (λ_ex_ = 507 nm, λ_em_ = 595 nm) on a Varioskan LUX microplate reader equipped with SkanIt Software for Microplate Readers RE, v5.0.0.42 (Thermo Fisher Scientific).

**Drug treatment, and cell viability (metabolic activity) and apoptosis assays**

For viability and AnnexinV assays, human cell lines were seeded at 100 cells/µL and incubated with the indicated concentrations of araC, vincristine, or regorafenib for 2 days, or of DNR for 1 day. Murine leukemic cells were seeded at 100/µL and incubated with the indicated concentrations of DNR or doxorubicin for 2 days. Regorafenib was from MCE, Monmouth Junction, NJ, USA; all other drugs were provided by the dispensary of the General Hospital, Vienna, Austria.

Cell viability was measured in white-walled 96-well-plates (Greiner Bio-One, Kremsmuenster, Austria) using the CellTiter-Glo® Luminescent Cell Viability Assay (Promega) and the Varioskan LUX microplate reader. To quantify the proportions of apoptotic cells after drug treatment, cells were stained with 2 µL of AnnexinV-APC (BD Biosciences) in 100 µL AnnexinV binding buffer (10 mM HEPES (Sigma-Aldrich), pH 7.4, 140 mM NaCl, 2.5 mM CaCl_2_ (Sigma-Aldrich)) for 15 min at room temperature, and analyzed by flow cytometry (LSR Fortessa, BD Biosciences). AnnexinV^-^ cells were classified as viable and AnnexinV^+^ as apoptotic. To assess activation of caspase-3, cells were seeded at 300/µL and incubated between 0 and 10 h with 2 µM araC, or between 0 and 12 h with 1 µM DNR. Samples were collected at the indicated time points, and protein extracts were prepared and subjected to immunoblot analysis as described above.

To investigate the effects of araC and DNR on *MTSS1* expression, HNT-34, TF-1, and KG-1 cells were seeded at 100/µL and incubated with IC_50_ concentrations of araC (13, 300, or 300 nM, respectively) for 2 days, or of DNR (150 nM, 600 nM, or 250 nM, respectively) for 1 day. RNA and protein preparation, as well as qRT-PCR and immunoblot analysis were carried out as described above.

**Drug screen**

A robot-assisted drug screen was performed on TF-1_sgMTSS1_1_C1, TF-1_sgMTSS1_2_C4, TF-1_sgCtrl_C1, and TF-1_sgCtrl_C2 cells, using a previously described library comprising 106 different compounds [9]. Most of these are approved for oncological indications, and they represent 13 different drug classes (Supplementary Table S1). Drugs were dissolved in DMSO, and for each of them, eight concentration steps of a 1:3 dilution series were tested in triplicate. 50 nL drug solution were plated into white-walled 384-well-plates using an Echo 550 (Labcyte, CA, USA), and 2 500 cells in 50 µL growth medium were added by a Multidrop™ Combi Reagent dispenser (Thermo Fisher Scientific). DMSO served as negative control, and 10 µM bortezomib as positive control. After 48 h, viability was measured using the CellTiter-Glo® Luminescent Cell Viability Assay. For the 52 drugs whose highest tested concentration inhibited the viability of the control clones by ≥ 50% (mean of both clones), area-under-the-curve (AUC) values were compared between knock-out and control clones. An AUC difference > 0.1 between the means of the knock-out and of the control clones was considered to indicate *MTSS1*-regulated sensitivity to that drug (Supplementary Table S1).

**RNA sequencing**

RNA sequencing (RNA-seq) was performed on TF-1 knock-out and control cells. For each of four different *MTSS1*-targeting sgRNAs (sgMTSS1_1, sgMTSS1_2, sgMTSS1_3, and sgMTSS1_4), four single cell clones with an immunoblot-confirmed knock-out were pooled, grown logarithmically overnight, and subjected to RNA extraction using TRIzol® reagent. For sgCtrl, three different pools of four clones each were processed similarly. RNA quality was measured on a Bioanalyzer (Agilent Technologies, Santa Clara, CA, USA.). To prevent distortions in gene expression values due to overcycling, the optimal number of PCR cycles for the library preparation was determined using the PCR Add-on kit (Lexogen, Vienna, Austria). RNA-seq libraries were prepared using the QuantSeq 3’ mRNA-Seq Library Prep Kit FWD for Illumina (Lexogen). Samples were submitted to the Next Generation Sequencing core facility of the Vienna BioCenter, Vienna, Austria, for 50 bp single-end sequencing. Unmapped .bam files were converted to .fastq format using SAMtools v1.10. Adapter trimming was performed using BBduk of BBMap v38.70, and sequence quality was checked using FASTQC v0.11.9. Reads were aligned to the human genome build GRCh38 using HISAT2 v2.1.0 with optimized settings for improved alignment rates [10]. Non-uniquely mapped reads were eliminated with SAMtools, and the remaining reads were used to identify differentially expressed genes with DESeq2 in R v3.6.1. An outlier test was performed based on the Mahalanobis distance (R package ClassDiscovery). For each sample of a group, distances (and their associated p-values) from the calculated center of all samples of this group were computed in a 2-dimensional principal component space. sgCtrl pool 3 and the sgMTSS1_4 pool had p-values < 0.05 and were excluded from further analyses. RNA-seq data were deposited in the Gene Expression Omnibus, acc. no. GSE152239.

**Congenic mouse model**

The effects of *Mtss1* on leukemogenesis *in vivo* were characterized in a C57BL/6 based, *MLL-AF9*-driven mouse model of AML [11-13]. In order to moderate disease aggressiveness, leukemic cells (LCs) from mice transplanted with pMSCV_*MLL-AF9*_IRES_Venus [14] transduced common myeloid progenitor cells (CMPs) were used (LC^MLL-AF9^). Spleen LC^MLL-AF9^ were transduced with pRRL-SFFV-GFP-mirE-PGK-NeoR containing shCtrl, shMtss1_1, or shMtss1_2 as described above, and sorted for Venus and GFP positivity on a BD FACSAria™ Fusion cell sorter (BD Biosciences, Franklin Lakes, NJ, USA). For transplantation, 6-8 week old female C57BL/6 mice were sub-lethally irradiated (5 Gy). On the next day, they were anaesthetized, and injected retro-orbitally with 500 000 transduced, Venus^+^ GFP^+^ LC^MLL-AF9^. Terminally ill mice were sacrificed and peripheral blood, BM, and spleen cells were collected. White and red blood cells and platelets were counted using a hematology analyzer Sysmex XN-350 (Sysmex, Kobe, Japan).

For flow cytometry experiments, vitally frozen BM LCs were thawed and recovered for 2 days in culture medium. Leukemic burden was defined as the proportion of Venus^+^ GFP^+^ cells among all viable cells. To assess myeloid differentiation, 500 000 recovered BM cells were washed with PBS and stained with 1 µL each of the Gr1 and CD11b antibodies (Supplementary Table S5) in 100 µL 2% FBS/PBS for 30 min. Gr1^+^ and Gr1^-^ cells represent more and less mature populations, respectively, among total myeloid leukemic (CD11b^+^ Venus^+^ GFP^+^) cells.

**Analyses of publicly available gene expression data sets**

The following Affymetrix gene expression microarray data sets were retrieved from the Gene Expression Omnibus database: GSE12417, GSE37642, GSE6891, and GSE1159. Raw data were processed, normalized, and log_2_ transformed using the frozen robust multiarray (R package fRMA) algorithm as described previously [13]. Samples with unknown cytogenetic status and with FAB type M3 were excluded. Differences between groups were calculated using the lmfit function of the limma package, which implements multiplicity correction for all probe sets present on an array, and FDR values < 0.05 were considered statistically significant. Group comparisons were visualized using the ggpubr package. Optimal cut-offs for classification of patients into MTSS1^high^ and MTSS1^low^ groups were calculated using maximally selected rank statistics (R package maxstat). The Kaplan Meier method was used to estimate survival distributions, and the log-rank test was applied to evaluate the statistical significance of differences in overall survival between MTSS1^high^ and MTSS1^low^ groups. P-values from these analyses were adjusted for multiple testing as described previously, and an adjusted p-value of < 0.05 was considered statistically significant [15]. The R packages survival and survminer were used for these calculations and for data visualization.

All genes that were significantly differentially expressed between TF-1 cells with or without knock-out of *MTSS1* were tested for correlation with *MTSS1* in data sets GSE1159, GSE12417, and GSE6891. A correlation coefficient exceeding ± 0.25 and an FDR < 0.05 in at least two of the three AML data sets, as well as consistency between the directions of the differential expression in the RNA-seq data and of the correlation in the AML data sets were used as criteria to define genes correlated with *MTSS1*.

All statistical tests were performed using R 3.4.2.

**Supplementary References**

1. Hackl H, Steinleitner K, Lind K, Hofer S, Tosic N, Pavlovic S, et al. A gene expression profile associated with relapse of cytogenetically normal acute myeloid leukemia is enriched for leukemia stem cell genes. Leuk Lymphoma. 2015;56:1126-8.

2. Montague TG, Cruz JM, Gagnon JA, Church GM, Valen E. CHOPCHOP: a CRISPR/Cas9 and TALEN web tool for genome editing. Nucleic Acids Res. 2014;42:W401-7.

3. Labun K, Montague TG, Gagnon JA, Thyme SB, Valen E. CHOPCHOP v2: a web tool for the next generation of CRISPR genome engineering. Nucleic Acids Res. 2016;44:W272-6.

4. Ran FA, Hsu PD, Wright J, Agarwala V, Scott DA, Zhang F. Genome engineering using the CRISPR-Cas9 system. Nat Protoc. 2013;8:2281-308.

5. Fellmann C, Hoffmann T, Sridhar V, Hopfgartner B, Muhar M, Roth M, et al. An optimized microRNA backbone for effective single-copy RNAi. Cell Rep. 2013;5:1704-13.

6. Livak KJ, Schmittgen TD. Analysis of relative gene expression data using real-time quantitative PCR and the 2(-Delta Delta C(T)) Method. Methods. 2001;25:402-8.

7. Utikal J, Gratchev A, Muller-Molinet I, Oerther S, Kzhyshkowska J, Arens N, et al. The expression of metastasis suppressor MIM/MTSS1 is regulated by DNA methylation. Int J Cancer. 2006;119:2287-93.

8. Kumaki Y, Oda M, Okano M. QUMA: quantification tool for methylation analysis. Nucleic Acids Res. 2008;36:W170-5.

9. Boidol B, Kornauth C, van der Kouwe E, Prutsch N, Kazianka L, Gultekin S, et al. First-in-human response of BCL-2 inhibitor venetoclax in T-cell prolymphocytic leukemia. Blood. 2017;130:2499-503.

10. Baruzzo G, Hayer KE, Kim EJ, Di Camillo B, FitzGerald GA, Grant GR. Simulation-based comprehensive benchmarking of RNA-seq aligners. Nat Methods. 2017;14:135-9.

11. Krivtsov AV, Twomey D, Feng Z, Stubbs MC, Wang Y, Faber J, et al. Transformation from committed progenitor to leukaemia stem cell initiated by MLL-AF9. Nature. 2006;442:818-22.

12. Nguyen CH, Bauer K, Hackl H, Schlerka A, Koller E, Hladik A, et al. All-trans retinoic acid enhances, and a pan-RAR antagonist counteracts, the stem cell promoting activity of EVI1 in acute myeloid leukemia. Cell Death Dis. 2019;10:944.

13. Nguyen CH, Gluxam T, Schlerka A, Bauer K, Grandits AM, Hackl H, et al. SOCS2 is part of a highly prognostic 4-gene signature in AML and promotes disease aggressiveness. Sci Rep. 2019;9:9139.

14. Zuber J, Rappaport AR, Luo W, Wang E, Chen C, Vaseva AV, et al. An integrated approach to dissecting oncogene addiction implicates a Myb-coordinated self-renewal program as essential for leukemia maintenance. Genes Dev. 2011;25:1628-40.

15. Altman DG, Lausen B, Sauerbrei W, Schumacher M. Dangers of using "optimal" cutpoints in the evaluation of prognostic factors. J Natl Cancer Inst. 1994;86:829-35.

**Supplementary Table S1:** Drug screen. List of drugs, drug classes, concentrations used, maximal inhibition of cellular viability relative to dimethyl sulfoxide (DMSO), and area under the curve (AUC) values.

Table is provided in separate file.

**Supplementary Table S2:** Results of RNA-seq analysis. (A) Genes differentially expressed at a false discovery rate (FDR) < 0.05 and a log_2_-fold change (log_2_FC) > 1 or < -1 between *MTSS1* knock-out and control cells. (B) Gene Ontology analysis of differentially expressed genes N, number. (C) List of transcription factors whose previously reported targets were significantly enriched in the list of differentially expressed genes. (D) Genes that were significantly differentially expressed between TF-1 *MTSS1* knock-out and control cells and correlated with *MTSS1* in data sets GSE6891, GSE12417, and GSE1159. (E) List of transcription factors whose previously reported targets were significantly enriched in the gene list in (D). (B, C, E) Analyses were performed using GeneGo MetaCore.

Table is provided in separate file.

**Supplementary Table S3:** Clinical characteristics of AML patients whose samples were used in this study.

|  |  |  |  | **Diagnosis** | | |  | **Relapse** | |  |  |
| --- | --- | --- | --- | --- | --- | --- | --- | --- | --- | --- | --- |
| **Pat no** | **Sex** | **Age** | **FAB** | **Karyotype** | **WBC** | **% blasts PB** | **Response** | **WBC** | **% blasts PB** | **Months from Dx to Rel** | **OS** |
| 1 | m | 42 | M1 | 46, XY | 35.000 | 83 | CR | 49.670 | 57 | 12 | 12 |
| 2 | f | 47 | M5 | 46, XX | 33.860 | 13 | CR | 59.420 | 56 | 7 | 8 |
| 3 | f | 36 | M2 | 46, XX | 57.000 | 85 | CR | 127.000 | 81 | 13 | 13 |
| 4 | f | 45 | M2 | 46, XX | 99.000 | 83 | CR | 200.000 | 83 | 13 | 15 |
| 5 | f | 46 | M4 | 46, XX | 66.990 | 46 | CR | 122.600 | 77 | 10 | >19* |
| 6 | f | 68 | M1 | 46, XX | 109.000 | 93 | PR | 111.950 | 79 | 7 | 8 |
| 7 | f | 49 | M0/M1 | 47, XX + marker chromosome | 108.000 | 65 | CR | 12.140 | 80 | 4 | 6 |
| 8 | m | 68 | M0/M1 | 45~47, XY, der(7)t(7;11)(p13;q13) del(7)(q31)[cp17]/46,XY[2] | 29.830 | 48 | CR | 43.400 | 1** | 10 | 10 |

Samples 1-6 were used for expression analysis; samples 5, 7, and 8 were used for promoter methylation analysis.

m, male; f, female

FAB, French American British classification

WBC, white blood cell count per µL

PB, peripheral blood

CR, complete remission; PR, partial response

Dx, diagnosis; Rel, relapse.

OS, overall survival in months

* lost to follow up

** 47% blasts after Ficoll-enrichment

All samples were from PB.

**Supplementary Table S4:** Oligonucleotides used for cloning of sgRNAs, and sequences of shRNAs, qRT-PCR, and PCR primers.

| **Oligonucleotides for cloning of sgRNAs** | | | |
| --- | --- | --- | --- |
|  | **forward** | **reverse** | **Target** |
| **sgCtrl** | 5'-CACCGGTATAATACACCGCGCTAC-3' | 5'-AAACGTAGCGCGGTGTATTATACC-3' | *Renilla luciferase* |
| **sg*MTSS1*_1** | 5’-CACCACATGAAGGTAGGACGCCGG-3’ | 5’-AAACCCGGCGTCCTACCTTCATGT-3’ | *MTSS1* exon 1,  pos. +63 to +82 |
| **sg*MTSS1*_2** | 5’-CACCAAGGAATGCAGCGCGCTCGG-3’ | 5’-AAACCCGAGCGCGCTGCATTCCTT-3’ | *MTSS1* exon 1,  pos. +17 to +36 |
| **sg*MTSS1*_3** | 5'-CACCAGACCACGCAAAAGGTACAG-3' | 5'-AAACCTGTACCTTTTGCGTGGTCT-3' | *MTSS1* exon 5,  pos. +369 to +388 |
| **sg*MTSS1*_4** | 5'-CACCGAGAAGGAATGCAGCGCGCT-3' | 5'-AAACAGCGCGCTGCATTCCTTCTC-3' | *MTSS1* exon 1,  pos. +13 to +32 |
|  | Vector specific overhangs are underlined. Target positions (pos.) are indicated relative to the A of the ATG. | | |
|  |  |  |  |
| **shRNAs against human *MTSS1* (Sigma-Aldrich)** | | | |
|  | **shRNA sequence** | **vendor's ID** | **Target** |
| **shCtrl** | not disclosed by vendor | SHC202 | "no human or murine gene" |
| **shMTSS1_1** | 5'-CCGGT**GATCCATTCCACTCTATAAT**C TCGAGATTATAGAGTGGAATGGATCAT TTTTTG-3' | TRCN0000412415 (Clone ID: NM_014751.4-2863s21c1) | *MTSS1* 3’-UTR |
| **shMTSS1_2** | 5'-CCGGT**TGTAGGCAACTCGGAATATA** CTCGAGTATATTCCGAGTTGCCTACAA TTTTTTG-3' | TRCN0000416018 (Clone ID: NM_014751.4-2897s21c1) | *MTSS1* 3’-UTR |
|  | *MTSS1* specific sequences are shown in **bold blue** letters. | |  |

| **shRNAs against murine *Mtss1* (Fellmann *et al.* 2013 [5])** | | | |
| --- | --- | --- | --- |
|  | **97-mer** | **shRNA ID** | **Target** |
| **shCtrl** | TGCTGTTGACAGTGAGCGC**AGGAATTATAATGCTTATCTA**TAGTGAAG  CCACAGATGTATAGATAAGCATTATAATTCCTATGCCTACTGCCTCGGA | shRen.713 | *Renilla luciferase* |
| **shMtss1_1** | TGCTGTTGACAGTGAGCGC**CACGCTGAAGCTGCAGAA**GAATAGTGAA  GCCACAGATGTATTCTTCTGCAGCTTCAGCGTGTTGCCTACTGCCTCGGA | shMtss1.930 | *Mtss1* CDS |
| **shMtss1_2** | TGCTGTTGACAGTGAGCGC**ACAGGTCTTTGTAAAATTTTA**TAGTGAAG  CCACAGATGTATAAAATTTTACAAAGACCTGTATGCCTACTGCCTCGGA | shMtss1.2881 | *Mtss1* 3’-UTR |
|  | Target specific sequences are shown in **bold blue** letters. |  |  |

| **qRT-PCR and PCR primer** | | | |
| --- | --- | --- | --- |
| **Forward primer** | | **Reverse primer** | |
| ***Mtss1*_fwd** | 5’-AGCTGCCAACCAGTTGTCTAAT-3’ | ***Mtss1_*rev** | 5’-CCTGCATGGGACTCACTTGAT-3’ |
| ***B2m*_fwd** | 5’-CCTTCAGCAAGGACTGGTCT-3’ | ***B2m*_rev** | 5’-TGTCTCGATCCCAGTAGACG-3’ |
| ***MTSS1*_exon1_fwd** | 5’-TCTCGGAAGGTGAAATTCCTT-3’ | ***MTSS1*_exon_1_rev** | 5’-GTGAGCAGGTGACACTCCG-3’ |
| **MTSS1_*bis*_fwd** | 5’-ATTATAAGYGGGTTTTGGGTTAGG-3’ | **MTSS1_*bis*_rev** | 5’-CAAACCACCACTAATACCCACTA-3‘ |
| **5'miR-Xho1** | 5’-CAGAAGGCTCGAGAAGGTATATTG  CTGTTGACAGTGAGCG-3’ | **3'miR-EcoR1** | 5’-CTAAAGTAGCCCCTTGAATTCCGA  GGCAGTAGGCA-3’ |
| ***TP53*_fwd** | 5’-GCTGCTCAGATAGCGATGGT-3’ | ***TP53*_rev** | 5’-TCTCGGAACATCTCGAAGCG-3’ |
|  | Restriction sites are underlined. |  |  |

**Supplementary Table S5:** Antibodies used for immunoblot analysis (IB) and flow cytometry (FC).

| **Method** | **Target** | **Company** | **Conjugate/ fluorophor** | **Clone/ cat.no.** | **Dilution** |
| --- | --- | --- | --- | --- | --- |
| IB | Human MTSS1 | Cell Signaling Technology | - | D2H4L XP® | 1:2.000 |
|  | Human WEE1 | Cell Signaling Technology | - | D10D2 | 1:1.000 |
|  | Human CDK1* | Cell Signaling Technology | - | POH1 | 1:1.000 |
|  | Human p-CDK1* (Tyr15) | Cell Signaling Technology | - | 10A11 | 1:1.000 |
|  | Human GAPDH | Cell Signaling Technology | - | 14C10 | 1:30.000 |
|  | Human cleaved caspase-3 | Cell Signaling Technology | - | 5A1E | 1:1.000 |
|  | Rabbit IgG | Jackson ImmunoResearch | Horseradish peroxidase | 111035008 | 1:10.000 |
| FC | Human *γ*H2AX | Cell Signaling Technology | Alexa Fluor® 647 | 9720 | 1:50 |
|  | Isotype control | Cell Signaling Technology | Alexa Fluor® 647 | 2985 | 1:200 |
|  | Mouse Gr1 | Biolegend | PE | RB6-8C5 | 1:100 |
|  | Mouse CD11b | Biolegend | Alexa Fluor® 700 | M1/70 | 1:100 |

* CDK1 is referred to by its alternative name CDC2 by Cell Signaling Technology.

**SUPPLEMENTARY FIGURE LEGENDS**

**Supplementary Figure S1: Low *MTSS1* expression is associated with poor overall survival in AML.** Kaplan-Meier curves showing the association between expression of *MTSS1* (probe set 210360_s_at) and overall survival in the publicly available AML gene expression data sets GSE37642 (n = 379; age, 18-83 years; cytogenetically heterogeneous) and GSE6891 (n = 222; age, 15-60 years; cytogenetically heterogeneous). Optimal cut-offs for each data set were determined using maximally selected rank statistics. Significance was calculated using the log-rank test; multiplicity correction was performed according to Altman *et al*. 1994 [15].

**Supplementary Figure S2: *MTSS1* expression is regulated by promoter methylation and reduced in response to cytotoxic drugs.** (a) Methylation status of the *MTSS1* promoter in U-937, HL-60, K-562, HNT-34, TF-1, and KG-1 cells. Distribution of methylated CpGs in a ~650 bp region surrounding the transcriptional start site of *MTSS1* and comprising part of its CpG island was determined by bisulfite sequencing. Empty circles, unmethylated CpGs; filled circles, methylated CpGs. Numbers refer to the positions of the CpGs relative to the transcription start site. (b) MTSS1 protein levels in U-937, HL-60, K-562, HNT-34, TF-1, and KG-1 cells treated with or without 5-aza-2'-deoxycytidine (5-aza) for 4 days were determined by immunoblot analysis. GAPDH was used as a loading control. Left panel, representative blots. Right panel, quantification. MTSS1 levels were normalized to GAPDH and to a calibrator sample (untreated U-937 cells for MTSS1^low^ cell lines; untreated HNT-34 cells for MTSS1^high^ cell lines). Means + SEM, n = 3. (c) Methylation status of the *MTSS1* promoter in paired diagnosis (Dx) - relapse (Rel) samples from three patients with AML (P5, P7, P8). The experiment was performed and presented as in (a). ×, missing information. (d, e) HNT-34, TF-1, and KG-1 cells were treated with IC_50_ concentrations of araC (13, 300, or 300 nM, respectively) for 2 days, or of DNR (150 nM, 600 nM, or 250 nM, respectively) for 1 day. Untreated controls were kept in parallel cultures for 2 days. (d) *MTSS1* mRNA levels were quantified by qRT-PCR, and normalized to *ß-2-microglobulin* levels and to untreated controls using the ΔΔC_T_ method. Means + SEM, n = 3. (e) MTSS1 protein levels were determined by immunoblot analysis. GAPDH was used as loading control. Left, representative experiments; right, quantifications. MTSS1 protein levels were normalized to GAPDH and to untreated controls. Means + SEM, n = 3. (b, d, e) * p < 0.05, ** p < 0.01, *** p < 0.001, (b) two-way ANOVA followed by Bonferroni’s post-hoc test, (d, e) Student’s one-sample *t*-test.

**Supplementary Figure S3: *MTSS1* knock-out in an AML cell line reduces apoptosis in response to araC and DNR.** (a) Analysis of sgRNA-induced mutations. In TF-1_sgMTSS1_1_C1, heterozygous insertions were found in 2/4 gDNA derived PCR clones each. TF-1_sgMTSS1_2_C4 showed a homozygous 13 bp deletion in 4/4 gDNA derived PCR clones. Nucleotide positions are indicated relative to the transcription start site. CDS, coding sequence; black bar, exon 1; grey bar, intron 1; blue bars, sgRNA target sequences. (b) Knock-out of *MTSS1* in single cell clones of sgRNA-transduced TF-1 cells was confirmed by immunoblot analysis; GAPDH was used as loading control. C1-C4, clone numbers. The antibody recognizes an epitope close to the C-terminus of the protein that is encoded by all reported transcript variants. (c) TF-1_sgCtrl_C1, TF-1_sgCtrl_C2, TF-1_sgMTSS1_1_C1, TF-1_sgMTSS1_1_C2, TF-1_sgMTSS1_2_C3, and TF-1_sgMTSS1_2_C4 were seeded at equal densities, and metabolic activity as a proxy for viable cell numbers was determined daily for 4 days. (d) AnnexinV assay; representative experiment. TF-1-derived *MTSS1* knock-out and control cells were incubated with or without the indicated concentrations of araC or DNR for 2 or 1 days, respectively, and stained with AnnexinV. AnnexinV^-^ cells were considered viable, and AnnexinV^+^ cells apoptotic. (e) Caspase-3 cleavage; quantification. TF-1-derived *MTSS1* knock-out and control cells were incubated with 2 µM araC or 1 µM DNR for the indicated times. Cleaved caspase-3 signal was normalized to GAPDH and to sgCtrl_C1 after 10 (araC) or 12 (DNR) h of incubation. (c, e) Means + SEM, n = 3. * p < 0.05, ** p < 0.01, *** p < 0.001, two-way ANOVA followed by Bonferroni’s post-hoc test.

**Supplementary Figure S4: *MTSS1* does not affect DNR uptake.** TF-1-derived *MTSS1* knock-out and control cells were incubated with the indicated concentrations of DNR for up to 10 h. DNR autofluorescence was detected in the PerCP channel. (a) Quantification. MFI, mean fluorescence intensity. Means + SEM, n = 3. Two-way ANOVA did not reveal any significant differences. (b) Representative experiment.

**Supplementary Figure S5: Knock-out of *MTSS1* in TF-1 cells enhances DNA damage checkpoint activity.** (a, b) TF-1 knock-out and control cells were incubated with 150 nM araC for 24 h or with 600 nM DNR for 16 h, followed by a 5 h washout period. Untreated controls were kept in parallel cultures for 24 or 16 h, respectively. Cells were harvested at the indicated time points and stained with an antibody against the double strand break binding histone variant γH2AX. (a) Quantification (γH2AX minus isotype control signal). MFI, mean fluorescence intensity. Means + SEM, n = 3. * p < 0.05, ** p < 0.01, two-way ANOVA followed by Bonferroni’s post-hoc test. (b) Representative experiment. (c) Immunoblot analysis showing levels of WEE1, p-CDK1 (Tyr15), and total CDK1 in *MTSS1* knock-out and control cells. GAPDH was used as a loading control. Left panel, representative blots. Right panel, quantifications. WEE1 and CDK1 levels were normalized to GAPDH; p-CDK1 levels were normalized to CDK1. TF-1_sgCtrl_C1 cells were used as calibrator sample. Means + SEM, n = 3. * p < 0.05, one-way ANOVA followed by Dunnett’s Multiple Comparison test.

**Supplementary Figure S6: *MTSS1* knock-down does not affect proliferation, but enhances chemotherapy resistance of human AML cell lines.** (a, c, e) TF-1, HNT-34, and KG-1 derivative cell lines obtained through transduction with *MTSS1* or control shRNAs were seeded at equal densities, and metabolic activity as a proxy for viable cell numbers was determined on the indicated days. (a) TF-1, (c) HNT-34, (e) KG-1 derivative cell lines. Means ± SEM, n = 3. Two-way ANOVA did not reveal any significant differences. (b, d, f) TF-1, HNT-34, and KG-1 derivative cell lines were incubated with or without the indicated concentrations of araC for 2 days (left panels), or of DNR for 1 day (right panels), and metabolic activity was measured as a proxy for viability. (b) TF-1, (d) HNT-34, (f) KG-1 derivative cell lines. Means + SEM, n = 3. * p < 0.05, ** p < 0.01, *** p < 0.001, two-way ANOVA followed by Bonferroni’s post-hoc test.

**Supplementary Figure S7: Knock-out of *MTSS1* increases the resistance of AML cells to vincristine and regorafenib.** (a) TF-1-derived *MTSS1* knock-out and control cells were incubated with the indicated concentrations of vincristine or regorafenib for 2 days, and stained with AnnexinV. AnnexinV^-^ cells were considered viable, and AnnexinV^+^ cells apoptotic. Representative experiment. (b) TF-1-derived *MTSS1* knock-out and control cells were incubated with the indicated concentrations of vincristine or regorafenib for 2 days, and metabolic activity was measured as a proxy for cell viability. Means + SEM, n = 3. * p < 0.05, ** p < 0.01, *** p < 0.001, two-way ANOVA followed by Bonferroni’s post-hoc test.

**Supplementary Figure S8: sgMTSS1_3 and sgMTSS1_4 are functionally equivalent to previously used sgRNAs.** (a) Knock-out of *MTSS1* in single cell clones of TF-1 cells transduced with sgMTSS1_3 and sgMTSS1_4 was verified by immunoblot analysis; GAPDH was used as loading control. Negative control (neg. ctrl), HL-60 cells; positive control (pos. ctrl), TF-1 cells; C1-C4, clone numbers. (b) *MTSS1* knock-out and control clones were incubated with the indicated concentrations of araC for 2 days, and metabolic activity was determined as a proxy for viability. Means + SEM, n = 2. * p < 0.05, ** p < 0.01, *** p < 0.001, two-way ANOVA followed by Bonferroni’s post-hoc test.

**Supplementary Figure S9: *Mtss1 k*nock-down does not affect spleen weight or blood parameters in *MLL-AF9* driven murine AML.** (a) *Mtss1* expression in normal murine bone marrow (nBM) cells and in BM leukemic cells from *MLL-AF9* driven murine AML (LC^MLL-AF9^). n = 3; means ± SEM. (b) Down-regulation of *Mtss1* in shMtss1_1 and shMtss1_2 *vs*. shCtrl transduced LC^MLL-AF9^ was confirmed by qRT-PCR. n = 3; means ± SEM. (c) Spleen weight and (d) blood parameters of mice terminally ill after transplantation with shMtss1 or shCtrl transduced LC^MLL-AF9^. WBC, white blood cells; RBC, red blood cells; PLT, platelets. Means + SEM, n = 3 - 4 mice. (a) Student’s two-sided *t*-test. (b-d) One-way ANOVA followed by Bonferroni’s post-hoc test. (c-d) One-way ANOVA did not reveal any significant differences.

**Supplementary Figure S10: *Mtss1* knock-down alters leukemic burden, myeloid differentiation, and anthracycline resistance in *MLL-AF9* driven murine AML.** BM cells were harvested from mice terminally ill after transplantation with shMtss1 or shCtrl transduced LC^MLL-AF9^. Representative flow cytometry plots. (a) Leukemic burden (proportion of Venus^+^ GFP^+^ cells). (b) Myeloid differentiation (proportion of mature (Gr1^+^) cells among myeloid leukemic (CD11b^+^ Venus^+^ GFP^+^) cells). (c) Anthracycline induced apoptosis. Cells incubated with the indicated concentrations of DNR or doxorubicin (doxo) for 2 days were stained with AnnexinV. AnnexinV^-^ cells were considered viable, and AnnexinV^+^ cells apoptotic.

**Supplementary Figure S11: Mutational status of *TP53* in the human AML cell lines used in this study.** The region containing the mutational hotspots of *TP53* (exons 6 to 8) was amplified by RT-PCR, and purified PCR products were sequenced. Sequences were aligned against the wild-type sequence (*TP53*_wt). Matches to the wild-type sequence are indicated by white letters on black background; mutations are indicated by black symbols.


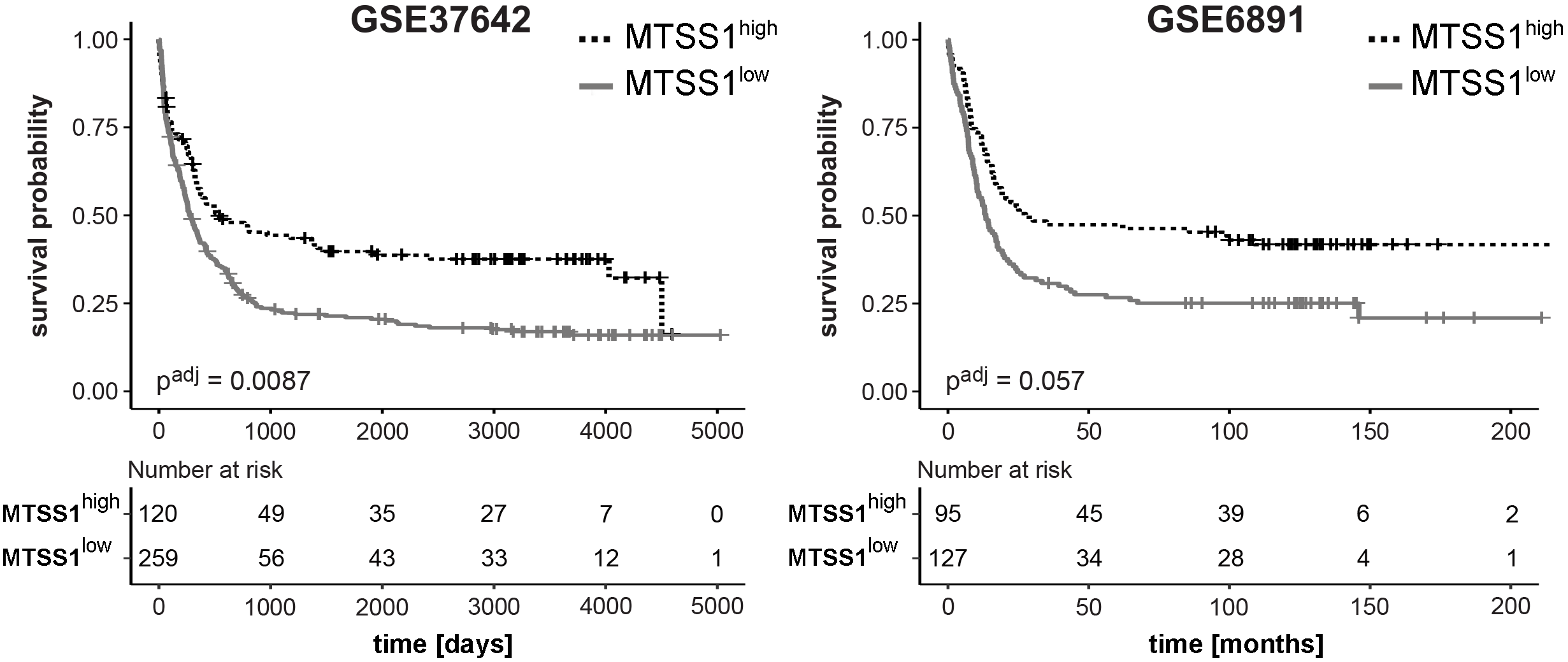


**Figure S1**

**
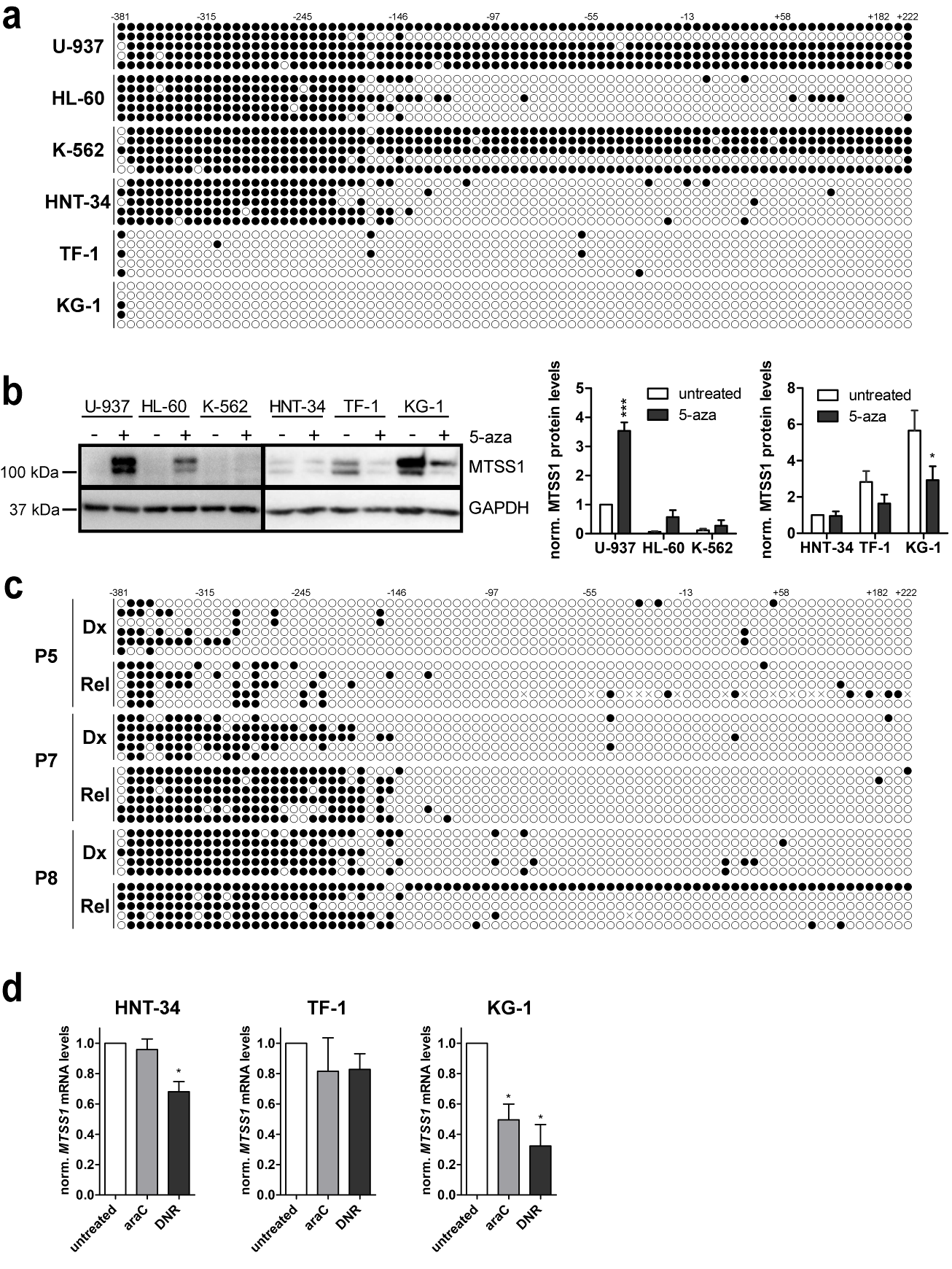
**

**Figure S2**

**
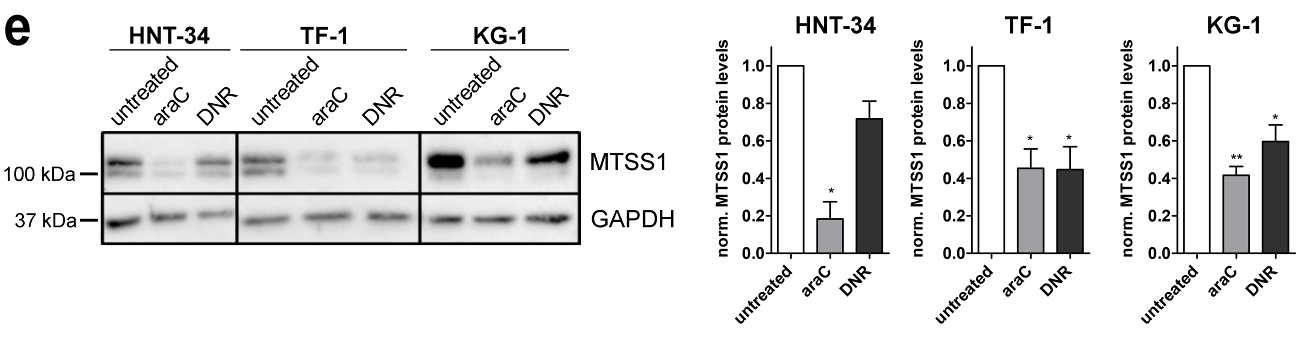
**

**Figure S2 – continued**

**
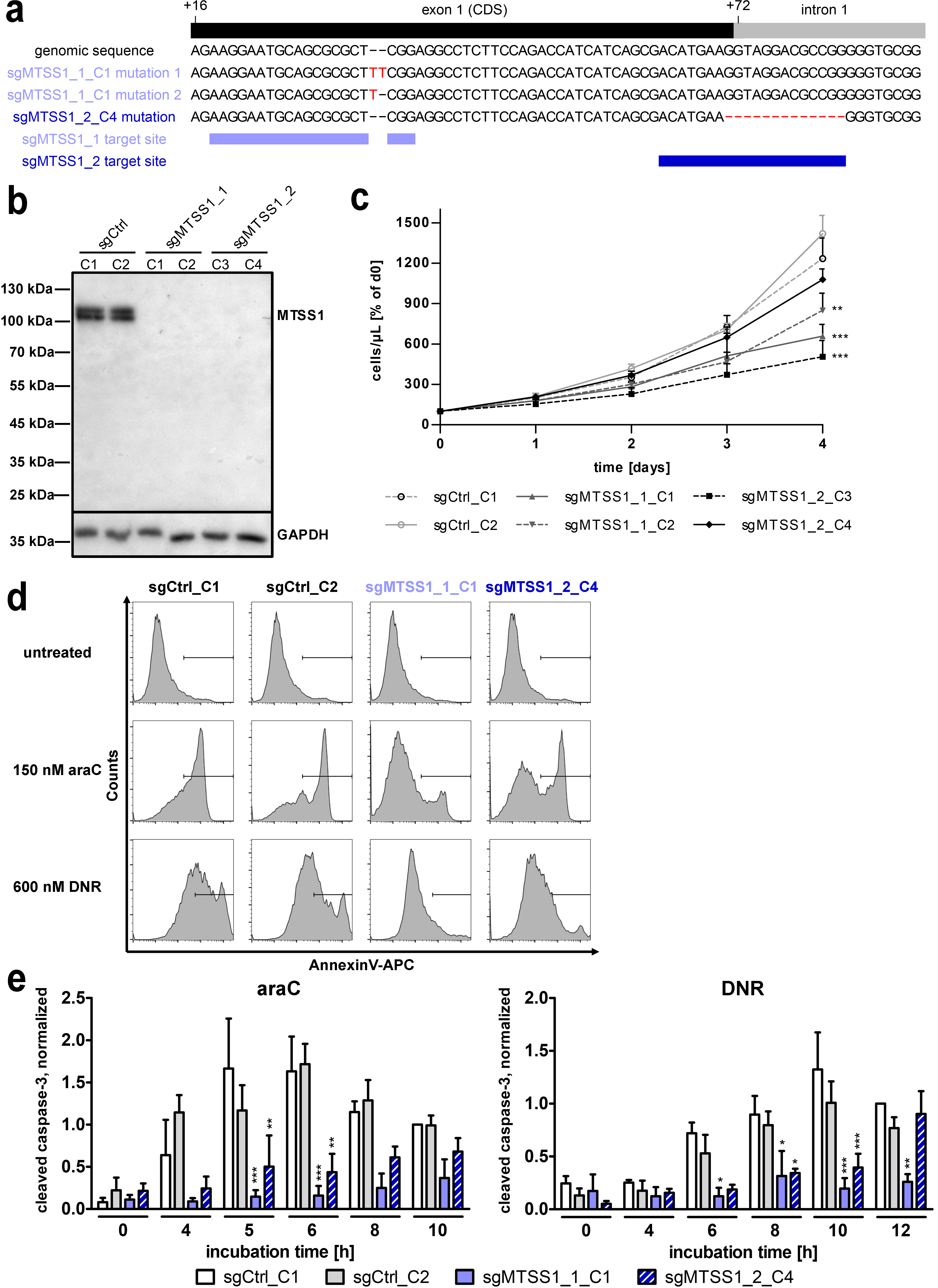
**

**Figure S3**

**
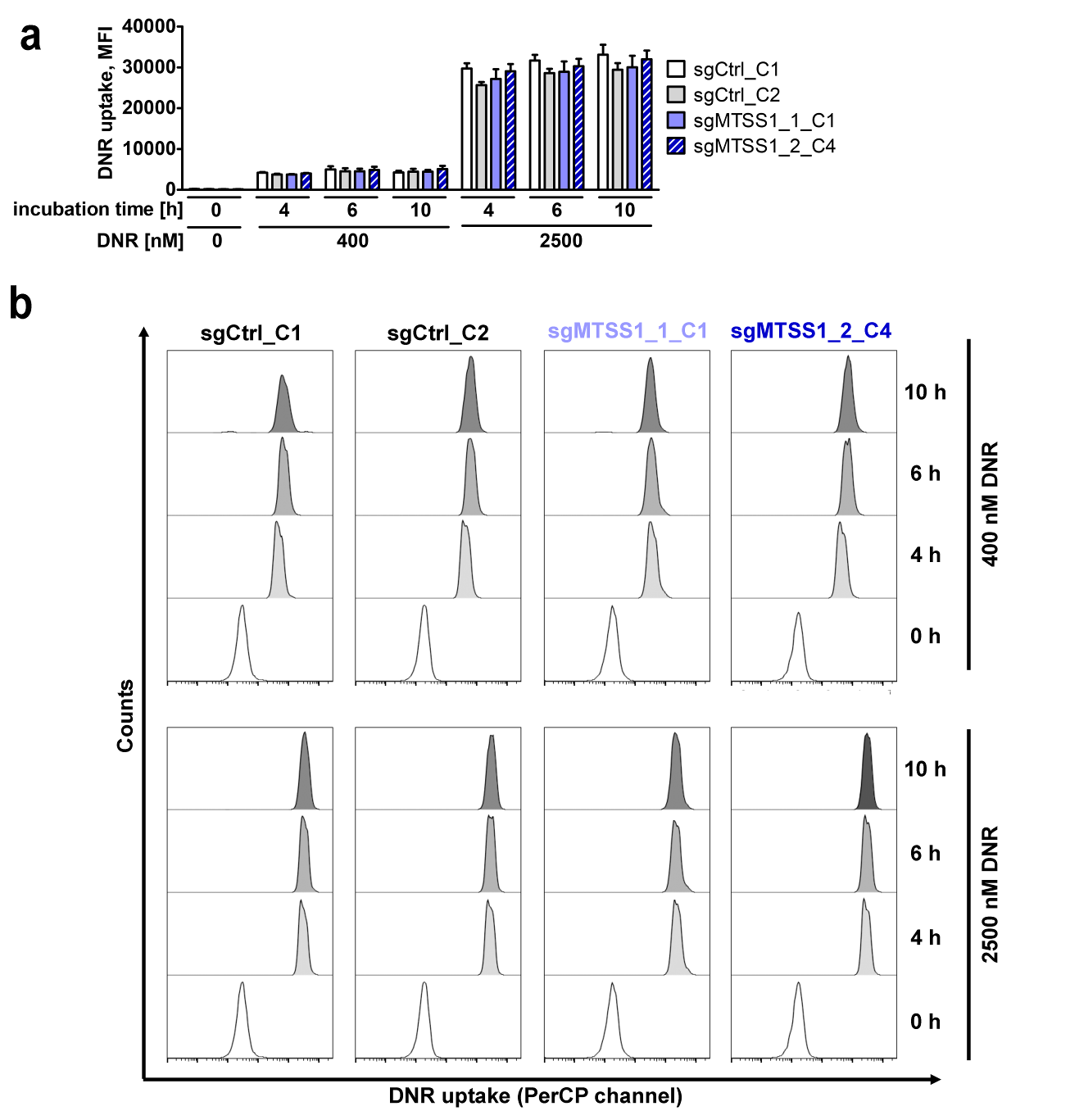
**

**Figure S4**

**
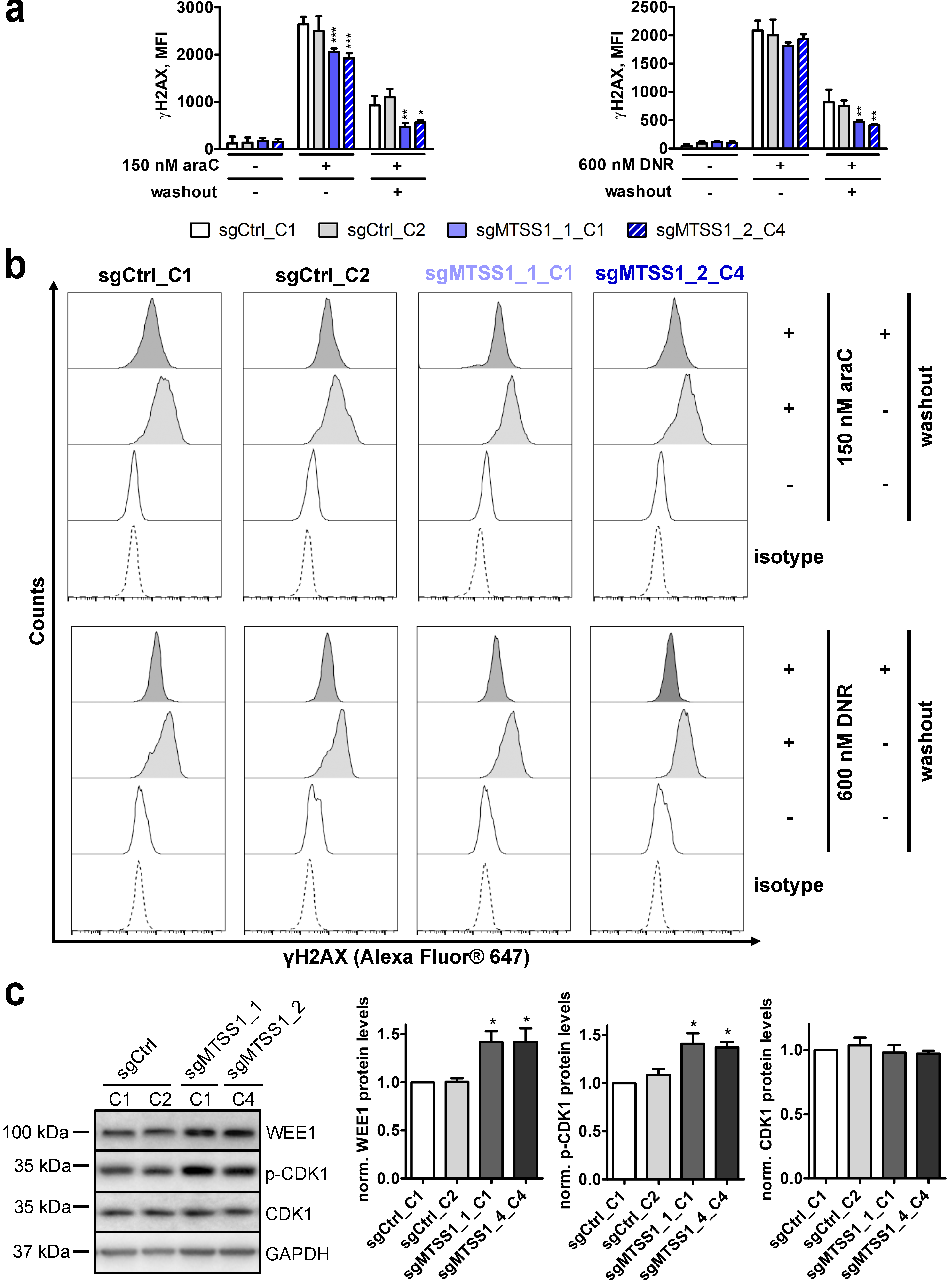
**

**Figure S5**

**
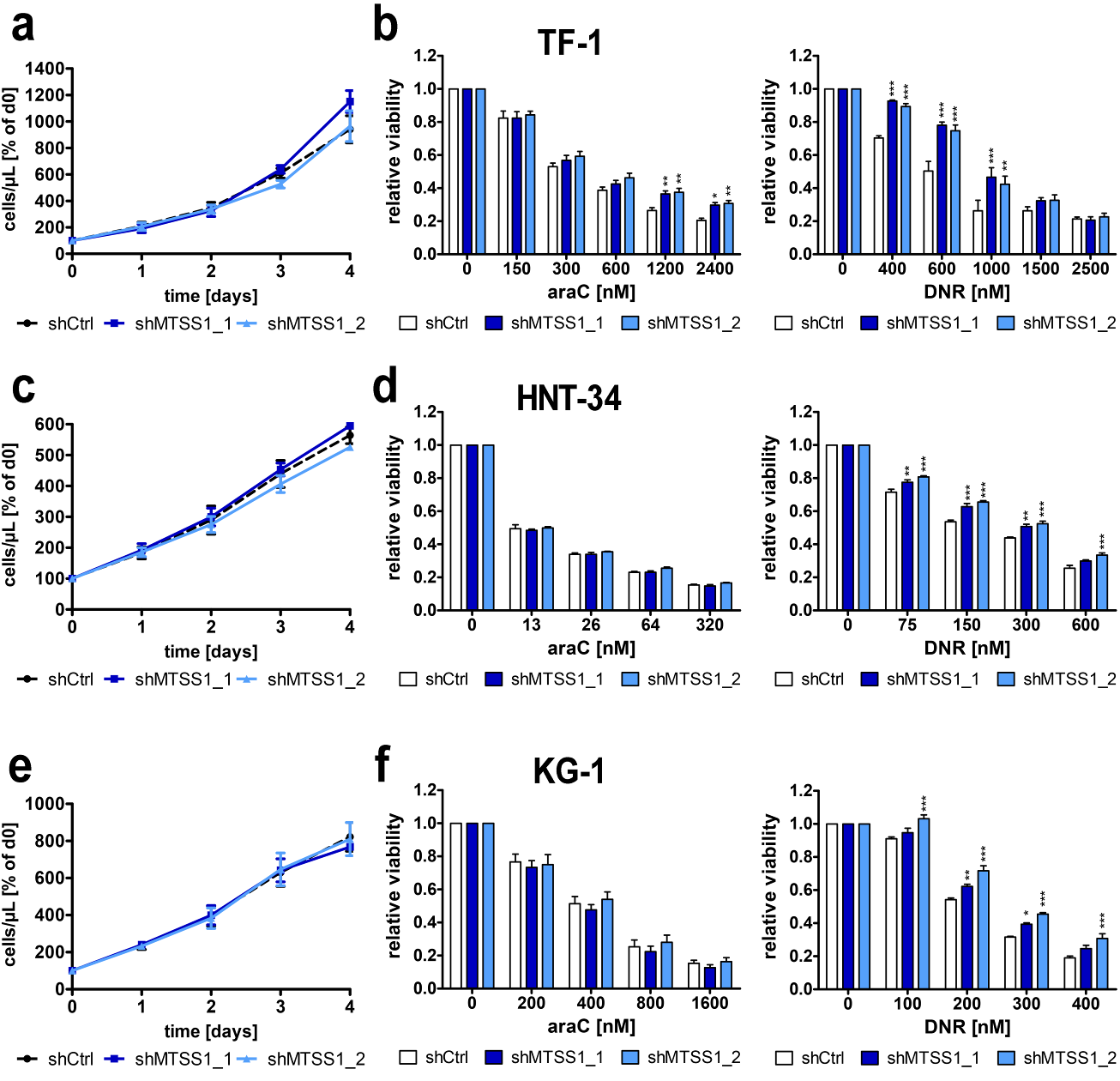
**

**Figure S6**

**
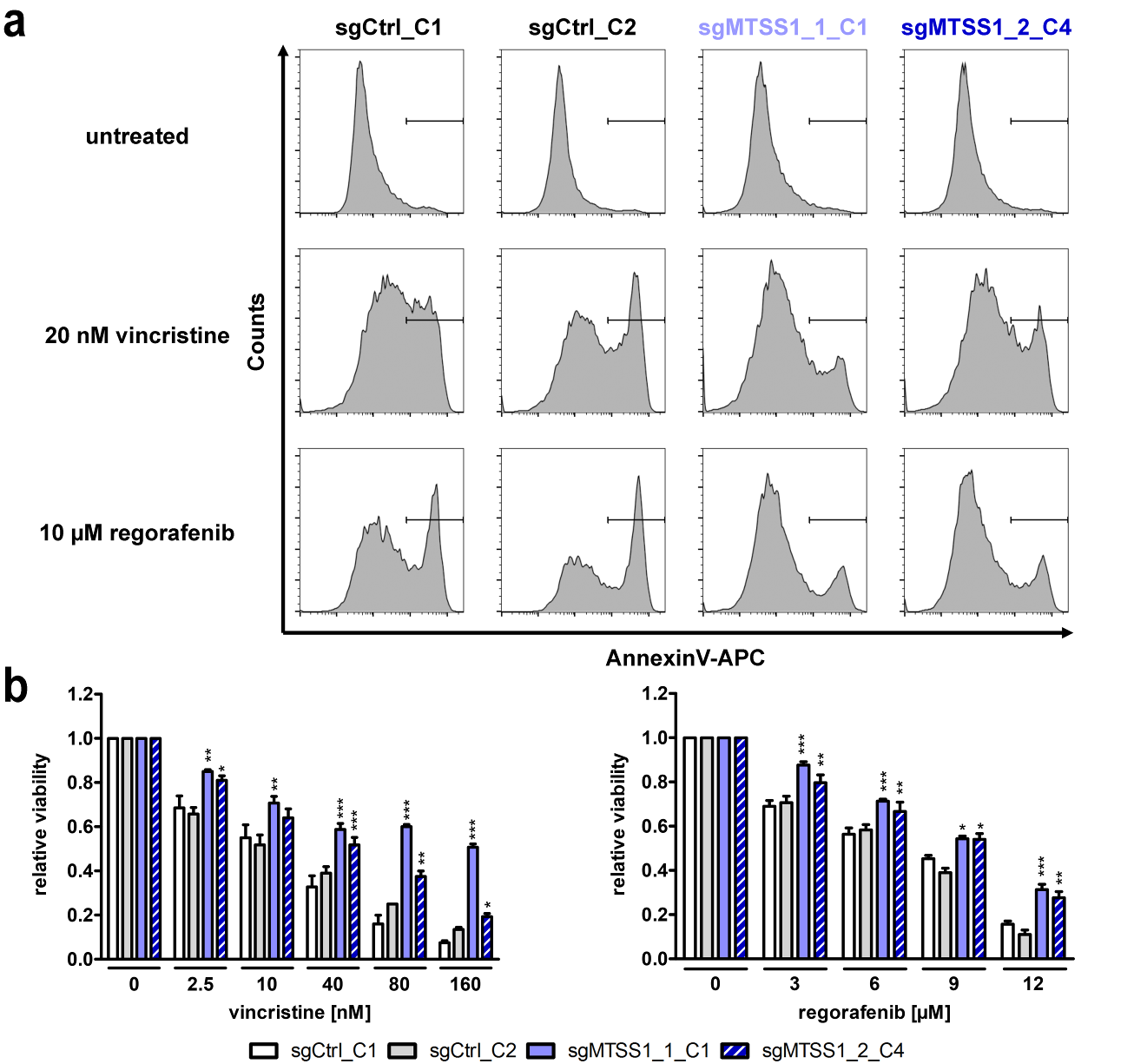
**

**Figure S7**

**
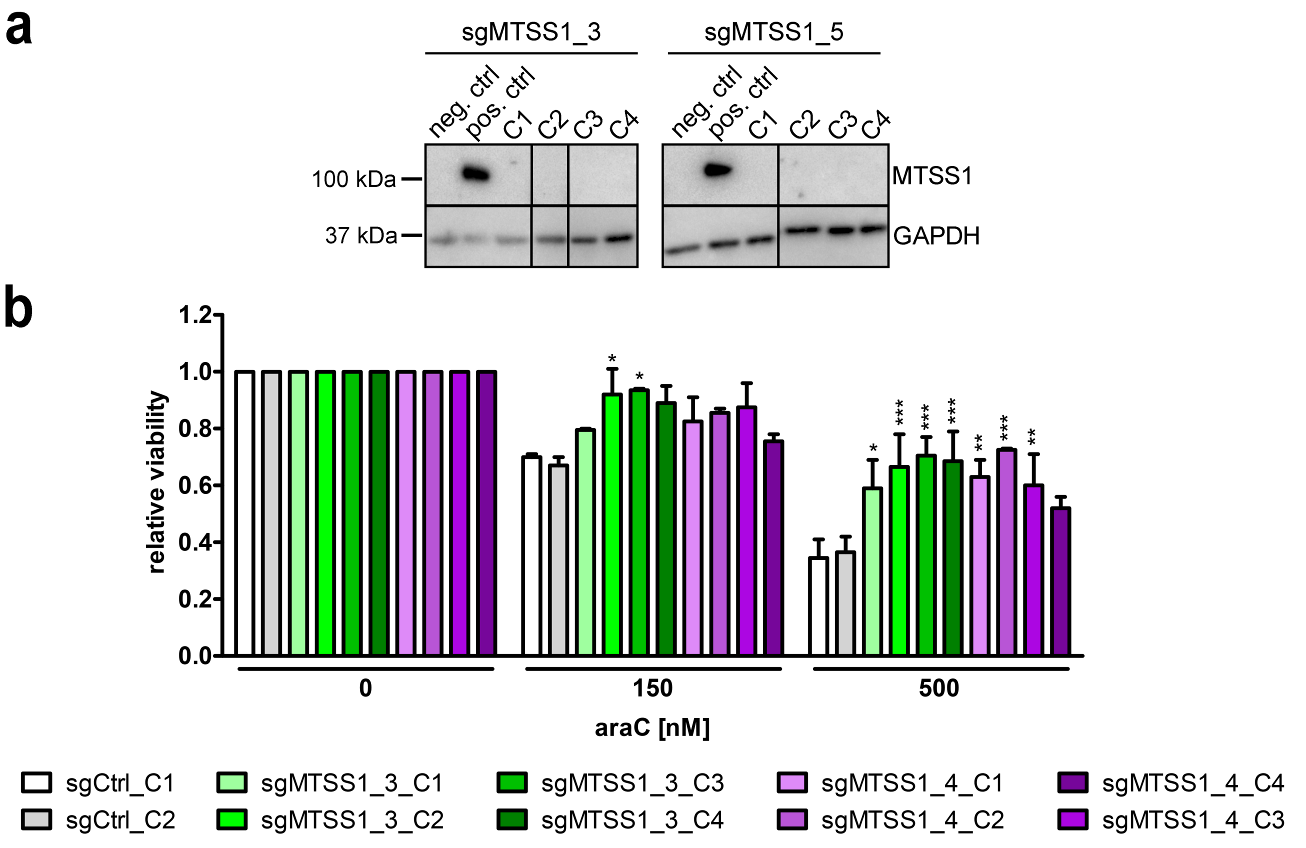
**

**Figure S8**

**
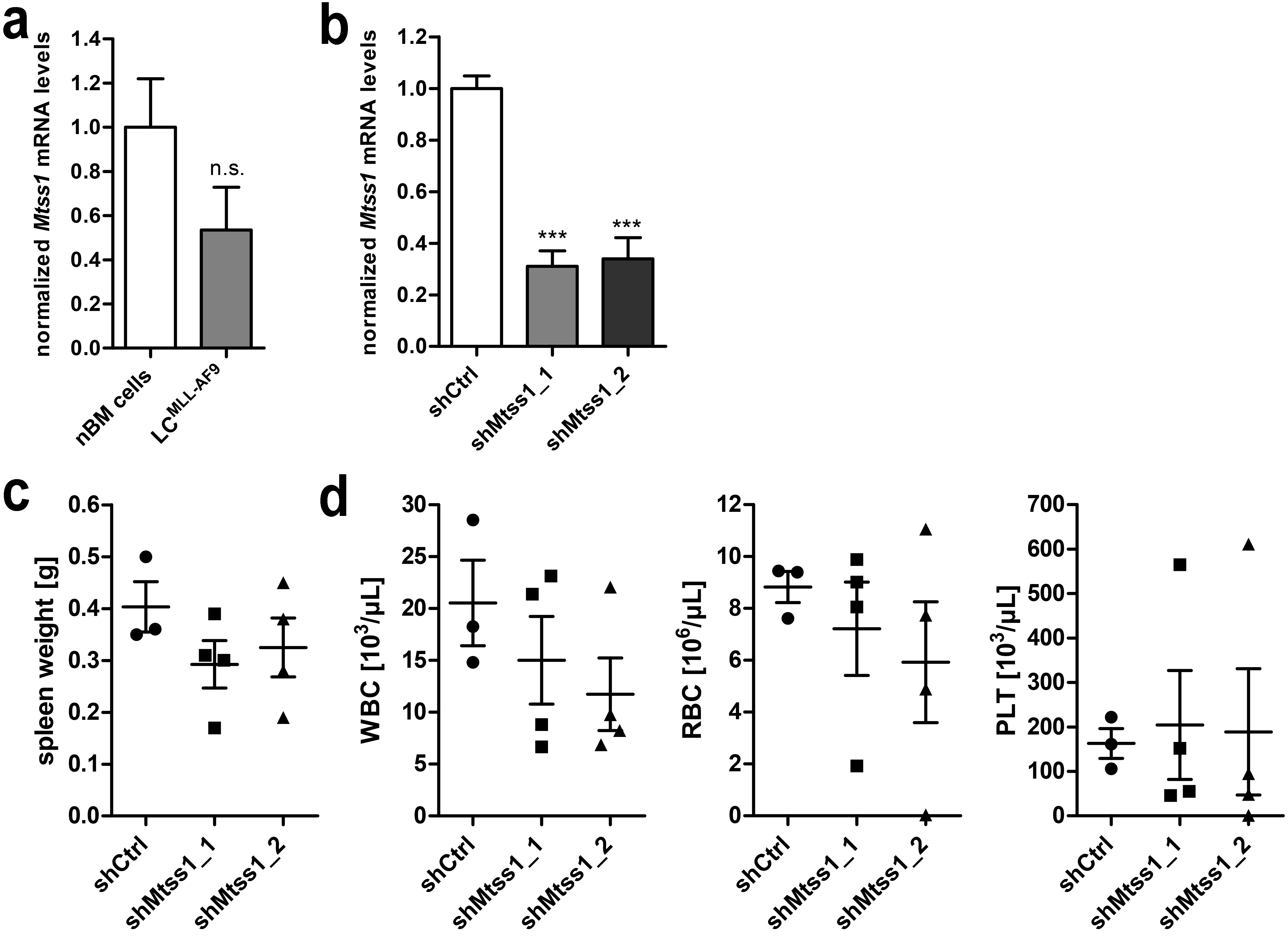
**

**Figure S9**

**
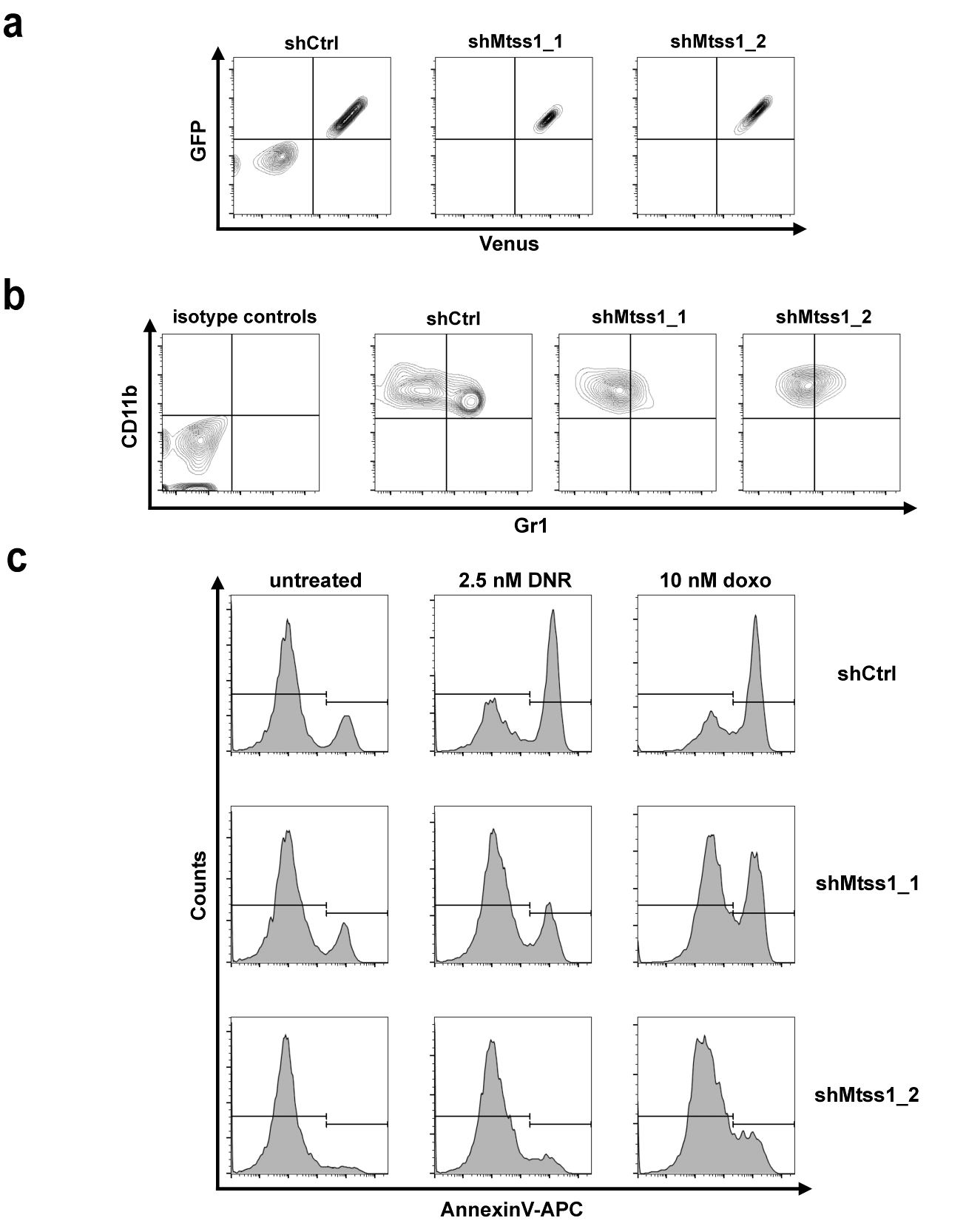
**

**Figure S10**

**
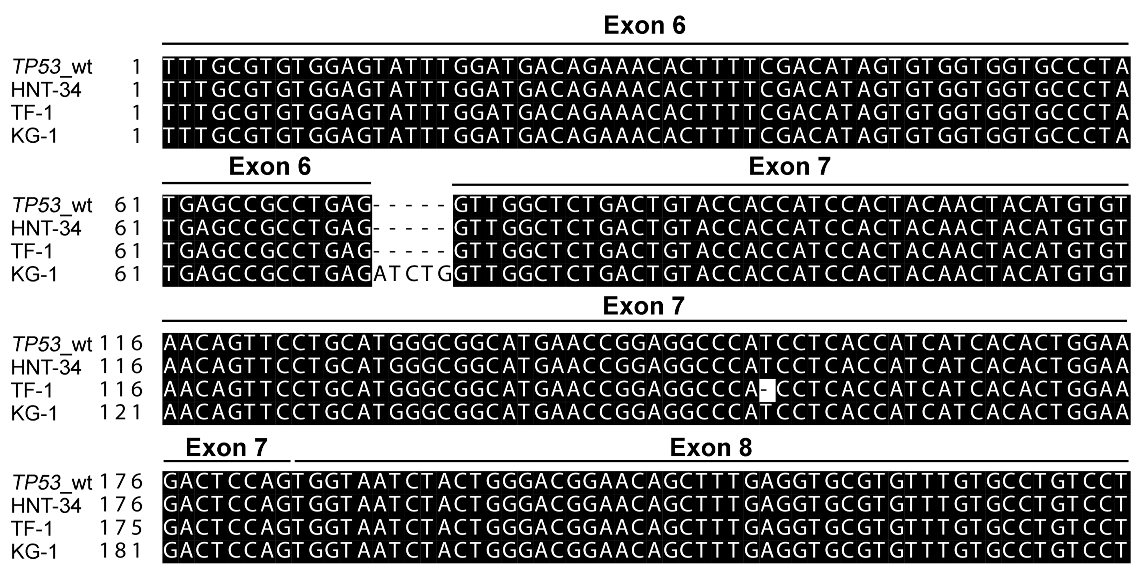
**

**Figure S11**
